# Genetic parameters, reciprocal cross differences, and age-related heterosis of egg-laying performance in chickens

**DOI:** 10.1186/s12711-023-00862-7

**Published:** 2023-12-07

**Authors:** Aixin Ni, Mario P. L. Calus, Henk Bovenhuis, Jingwei Yuan, Yuanmei Wang, Yanyan Sun, Jilan Chen

**Affiliations:** 1 State Key Laboratory of Animal Biotech Breeding, Key Laboratory of Animal (Poultry) Genetics Breeding and Reproduction of Ministry of Agriculture and Rural Affairs, Institute of Animal Science, Chinese Academy of Agricultural Sciences, Beijing, 100193 China; 2 Animal Breeding and Genomics, Wageningen University and Research, P.O. Box 338, 6700 AH Wageningen, The Netherlands

## Abstract

**Background:**

Egg-laying performance is economically important in poultry breeding programs. Crossbreeding between indigenous and elite commercial lines to exploit heterosis has been an upward trend in traditional layer breeding for niche markets. The objective of this study was to analyse the genetic background and to estimate the heterosis of longitudinal egg-laying traits in reciprocal crosses between an indigenous Beijing-You and an elite commercial White Leghorn layer line. Egg weights were measured for the first three eggs, monthly from 28 to 76 weeks of age, and at 86 and 100 weeks of age. Egg quality traits were measured at 32, 54, 72, 86, and 100 weeks of age. Egg production traits were measured from the start of lay until 43, 72, and 100 weeks of age. Heritabilities and phenotypic and genetic correlations were estimated. Heterosis was estimated as the percentage difference of performance of a crossbred from that of the parental average. Reciprocal cross differences were estimated as the difference between the reciprocal crossbreds as a percentage of the parental average.

**Results:**

Estimates of heritability of egg weights ranged from 0.29 to 0.75. Estimates of genetic correlations between egg weights at different ages ranged from 0.72 to 1.00. Estimates of heritability for cumulative egg numbers until 43, 72, and 100 weeks of age were around 0.15. Estimates of heterosis for egg weight and cumulative egg number increased with age, ranging from 1.0 to 9.0% and from 1.4 to 11.6%, respectively. From 72 to 100 weeks of age, crossbreds produced more eggs per week than the superior parent White Leghorn (3.5 eggs for White Leghorn, 3.8 and 3.9 eggs for crossbreds). Heterosis for eggshell thickness ranged from 2.7 to 6.6% when using Beijing-You as the sire breed. No significant difference between reciprocal crosses was observed for the investigated traits, except for eggshell strength at 54 weeks of age.

**Conclusions:**

The heterosis was substantial for egg weight and cumulative egg number, and increased with age, suggesting that non-additive genetic effects are important in crossbreds between the indigenous and elite breeds. Generally, the crossbreds performed similar to or even outperformed the commercial White Leghorns for egg production persistency.

**Supplementary Information:**

The online version contains supplementary material available at 10.1186/s12711-023-00862-7.

## Background

Heterosis refers to the phenomenon that performance of hybrids is superior to the average performance of their parental breeds or lines. Heterosis is reported to be caused by non-additive genetic effects [1] and is widely exploited in the breeding of commercial layers. There are growing trends in consumers’ preference for egg products from traditional breeds and crossbreeding between indigenous breeds and commercial elite breeds is an important method to meet this unique market preference. In China, eggs from crossbreds of indigenous chickens and elite lines have taken over 10% of the domestic market [2]. One of the major laying hen producers, Hendrix Genetics, has several specific layer lines for regional preference, e.g., Azur for bule eggshell colour, and Olive for small egg producers.

Indigenous Beijing-You chickens produce eggs with a higher yolk proportion and lecithin content than commercial layer breeds, such as White Leghorn [3], but they show lower egg production [4]. Crossing these two breeds is expected to manifest a high degree of heterosis because, first, the heritability for egg-laying traits is generally low to moderate [5–8] and heterosis is thought to be inversely related with heritability [9, 10], and second, these two breeds are genetically distant from each other [11]. A better understanding of heterosis for egg-laying performance may have implications for setting up more effective breeding schemes for layer chicken.

Several studies have estimated heterosis for egg weight [12], egg number [13–16], and egg quality [17]. Ledur et al. reported fluctuating levels of heterosis for egg number across various stages of the egg laying period [14]. Differences in heterosis at different ages were also observed for many traits, i.e., 2.5 and − 0.3% for egg weight at 26 and 59 weeks of age, respectively [12], and − 1.2 to 0.1% for Haugh unit at 35, 50, and 65 weeks of age [17]. Moreover, current breeding programs focus on prolonging the laying period up to 100 weeks of age. These results and recent developments emphasize the need for studies to measure egg-laying performance of crossbred layers throughout the laying period.

Some studies have investigated differences between reciprocal crosses [16, 18, 19], which are thought to result from maternal effects and sex-linked genes [20]. For instance, using indigenous Egyptian chickens as the sire line instead of the dam line led to later sexual maturity when it was crossed with a commercial Lohmann breed [16]. A better understanding of the reciprocal cross differences will contribute to a better decision about which breed should be the sire line in a crossbreeding scheme for a specific set of traits.

In the present study, purebreds and their reciprocal crosses of Beijing-You and White Leghorn were bred and monitored for egg weights, egg production, and egg quality traits, to analyse the genetic background and estimate heterosis of longitudinal economically important egg laying traits throughout the laying period.

## Methods

### Parental lines

The Beijing-You chickens (Y) used here were from a pedigreed pure line kept on the experimental farm of the Institute of Animal Science of the Chinese Academy of Agricultural Sciences. This line was selected for egg number and egg weight at a relatively low intensity for 18 generations. In the most recent five generations, about 60 roosters were mated with 500 hens, avoiding matings that would result in offspring with an inbreeding coefficient higher than 0.04. The other parental line, White Leghorn (W, Shaver line), was imported from the University of Guelph in 2018. The W chickens were kept on the same farm as the Y chickens for two generations using random mating. The Y and W chickens were kept in individual cages and fed ad libitum with a breeder diet containing 19% crude protein (CP), 2840 kcal/kg metabolizable energy (ME), 3.50% Ca, and 0.32% nonphytate P. The two breeds were mated to generate four genetic groups, using 30 Y and 30 W roosters, and 300 Y and 300 W hens: (1) 30 Y roosters with good sperm motility and sperm concentration were randomly mated with 150 Y hens to generate YY purebred offspring, (2) the same 30 Y roosters were randomly mated with 150 W hens to generate YW crossbred offspring, (3) 30 W roosters with good sperm motility and sperm concentration were randomly mated with 150 W hens to generate WW purebred offspring, and (4) the same 30 W roosters were randomly mated with 150 Y hens to generate WY crossbred offspring. In total, 1369, 1838, 1372, and 1836 eggs were collected for hatching for YY, WW, WY, and YW, respectively.

### Experimental genetic groups

In total, 487 YY, 675 WW, 507 WY, and 714 YW healthy female chicks were hatched simultaneously. They were vaccinated against Marek’s disease, infectious bursal, Newcastle disease, and infectious bronchitis, and transferred to the same brooding pen. At 18 weeks of age, 315 YY, 271 WW, 315 WY, and 359 YW chickens were randomly selected from all available chickens and transferred to the laying hens' house and kept in individual cages to measure egg-laying performance. Seven cages (length 37 cm × width 34 cm × height 34 cm) were stacked on one of three tiers in each bank; 15 banks were arranged in one row, for three rows in total. Some chickens from the same hatch were randomly selected and used for a different experiment. The average survival till 18 weeks was similar for the four genetic groups and ranged from 96.0 to 97.5%. Chickens were fed the same diet ad libitum and kept under the same controlled environmental conditions. The daily lighting increased by one hour per week from 8 h at 19 weeks of age to 13 h at 24 weeks of age, and increased by 0.5 h per week from 25 to 30 weeks of age. A constant daily lighting of 16 h was maintained thereafter.

### Egg-laying performance

#### Egg weight

The average weight of the first three eggs of each bird was calculated and will be referred to as the first egg weight. Egg weight was measured every four weeks from 28 to 76 weeks of age, and at 86 and 100 weeks of age. Egg weights were measured using a digital scale with a sensitivity of 0.01 g (HC-UTP-313, Haihua Chao Electrical Appliances, Shanghai, China).

#### Egg production

Age at first egg and cumulative egg number till 43, 72, and 100 weeks of age (EN43, EN72, and EN100) were computed from individual egg-laying recordings. The oviposition period, representing the average time interval between two successive layings was calculated following the method of Blake et al. [21]. Briefly, times of egg laying were recorded for each hen from the beginning of 31 weeks of age till the end of 33 weeks of age from 07:00 to 19:00 at 0.5-h intervals with a radio frequency identification recording system (Litrace Beijing Co., Ltd., Beijing, China). The oviposition period was calculated from the exact laying time of each egg for each hen. In addition, clutch-related traits were derived from the egg-laying recordings, including the number of clutches, average clutch length, and average pause length. These traits provide insight into the regularity of the laying of eggs over time. The number of clutches was computed as the number of times a hen had successive laying days preceded or followed by at least one day pause [22]. Average clutch length was calculated as the total egg number divided by the number of clutches within the observation period [22]. The number of pauses was computed as the number of clutches minus one. Average pause length was calculated as the total pause days between the first and last clutch divided by the number of pauses [23].

#### Egg quality

Egg quality traits were measured within 24 h after egg collection at 32, 54, 72, 86, and 100 weeks of age and included egg shape index, eggshell colour, eggshell strength, eggshell thickness, eggshell ratio, yolk colour, yolk ratio, and Haugh unit. The egg shape index was calculated as the ratio of egg width to egg length [24], which was measured using the FHK egg dimension meter (NFN385, Fujihira Ind. Co. Ltd., Tokyo, Japan). Eggshell colour was measured using a QCR-shell colour reflectometer (Technical Services and Supplies, England). Measurements were expressed as a percentage reading between black (0%) and white (100%). Eggshell strength, representing the force required to break the shell of an intact egg, was measured at the obtuse pole of the egg using the Egg Force Reader (Orka Food Technology Ltd., Israel). Eggshell thickness was measured at the acute, middle, and obtuse poles of each egg using the Eggshell Thickness Gauge (NFN380, Fujihira Ind. Co. Ltd., Tokyo, Japan), with a sensitivity of 0.01 mm. Yolk colour and Haugh unit were measured using the Egg Analyzer (Orka Food Technology Ltd., Israel). Egg weight, eggshell weight, and yolk weight were measured using a digital scale with a sensitivity of 0.01 g (HC-UTP-313). Eggshell ratio and yolk ratio were computed as the ratio of eggshell weight and yolk weight, respectively, to egg weight.

### Statistical analyses

For egg production traits, chickens that did not lay any eggs (6 WW, 8 WY, 11 YW, and 7 YY) were removed from the dataset. Subsequently, outliers were removed by excluding data that deviated more than three standard deviations from the mean of their genetic group (see Additional file 1: Tables S1, S2, and S3 for percentage of outliers removed). Descriptive statistics were calculated using the “pastecs” package in R software (https://www.r-project.org/).

For egg production traits with only one observation, pedigree-based heritabilities were estimated with the average information restricted maximum likelihood (AI-REML) method in ASReml 4.2 [25] using the following univariate model:

1 $$\mathbf{y}= \mathbf{1}\mu +{\mathbf{X}}_{1}\mathbf{b}+{\mathbf{X}}_{2}\mathbf{r}+\mathbf{Z}\mathbf{a}+\mathbf{e},$$

where $\mathbf{y}$ is the vector of phenotypic values across the four genetic groups (YY, YW, WW, and WY), $\mathbf{1}$ is a vector of ones, $\mu$ is the population average, $\mathbf{b}$ is the vector of the fixed effect of genetic group, ${\mathbf{X}}_{\mathbf{1}}$ is the design matrix for genetic groups, $\mathbf{r}$ is the vector of the fixed effects of the rack (24 levels for different tiers, rows, and banks), ${\mathbf{X}}_{\mathbf{2}}$ is the design matrix for rack effects, $\mathbf{a}$ is the vector of the random animal effects with $N(\mathbf{0},\mathbf{A}{\sigma }_{a}^{2})$, where $\mathbf{A}$ is the pedigree-based relationship matrix and ${\sigma }_{a}^{2}$ is the additive genetic variance, $\mathbf{Z}$ is the design matrix that relates phenotypes to additive genetic effects, $\mathbf{e}$ is the vector of random residual effects, with a separate residual variance assumed for each genetic group, e.g., ${\mathbf{e}}_{\mathbf{W}\mathbf{W}} \sim N(\mathbf{0},{\mathbf{I}}_{\mathbf{W}\mathbf{W}}{\sigma }_{{e}_{WW}}^{2})$ for the WW genetic group, where ${\mathbf{I}}_{\mathbf{W}\mathbf{W}}$ is the identity matrix and ${\sigma }_{{e}_{WW}}^{2}$ is the residual variance for the WW genetic group. Finally, ${\sigma }_{e}^{2}$ was computed as the average residual variance over the four genetic groups. To build the $\mathbf{A}$ matrix, the pedigree depth of YY was trimmed to five generations using the R-package “pedigree” [26]. For egg production traits, heritability (${h}^{2}$) was computed as:

$${h}^{2}=\frac{{\sigma }_{a}^{2}}{{\sigma }_{a}^{2}+{\sigma }_{e}^{2}}.$$

For egg weight and egg quality traits, which had multiple observations, the phenotypic data was analysed using Model (1) extended with a permanent environmental effect:

2 $$\mathbf{y}={\mathbf{1}\mu +\mathbf{X}}_{\mathbf{1}}\mathbf{b}+{\mathbf{X}}_{2}\mathbf{r}+\mathbf{Z}\mathbf{a}+\mathbf{W}\mathbf{p}\mathbf{e}+\mathbf{e},$$

where effects are the same as described for Model (1) and $\mathbf{p}\mathbf{e}$ is the vector of random permanent environmental effects, with $N(\mathbf{0},\mathbf{I}{\sigma }_{pe}^{2})$, where ${\sigma }_{pe}^{2}$ is the permanent environmental variance, and $\mathbf{W}$ is the incidence matrix linking phenotypes to permanent environmental effects. For these traits, heritability (${h}^{2}$) and repeatability ($r$) were estimated as:

$${h}^{2}=\frac{{\sigma }_{a}^{2}}{{\sigma }_{a}^{2}+{\sigma }_{pe}^{2}+{\sigma }_{e}^{2}},$$

$${\text{and}}\, r=\frac{{\sigma }_{a}^{2}+{\sigma }_{pe}^{2}}{{\sigma }_{a}^{2}+{\sigma }_{pe}^{2}+{\sigma }_{e}^{2}}.$$

Phenotypic and genetic correlations between traits, as well as within traits measured at different ages, were estimated using bivariate models. The fixed effects were the same as for the univariate models. The additive genetic effects were assumed to be distributed as:

$$N\left(\left[\begin{array}{c}\mathbf{0}\\ \mathbf{0}\end{array}\right], \mathbf{A}\otimes \left[\begin{array}{cc}{\sigma }_{a, T1}^{2}& {r}_{a}{\sigma }_{a,T1}{\sigma }_{a,T2}\\ {r}_{a}{\sigma }_{a, T1}{\sigma }_{a,T2}& {\sigma }_{a,T2}^{2}\end{array}\right]\right),$$

where ${\sigma }_{a, T1}^{2}$ (${\sigma }_{a,T2}^{2}$) is the additive genetic variance of trait 1 (trait 2) and ${r}_{a}$ is the additive genetic correlation between traits 1 and 2, and $\otimes$ is the Kronecker product. The residuals for the two traits were assumed distributed as:

$$N\left(\left[\begin{array}{c}\mathbf{0}\\ \mathbf{0}\end{array}\right],\boldsymbol{ }\mathbf{I}\otimes \left[\begin{array}{cc}{\sigma }_{e, T1}^{2}& {r}_{e}{\sigma }_{e,T1}{\sigma }_{e,T2}\\ {r}_{e}{\sigma }_{e, T1}{\sigma }_{e,T2}& {\sigma }_{e,T2}^{2}\end{array}\right]\right),$$

where ${\sigma }_{e, T1}^{2}$ (${\sigma }_{e,T2}^{2}$) is the residual variance of trait 1 (trait 2) and ${r}_{e}$ is the residual correlation between traits 1 and 2.

In the bivariate version of Model (2), permanent environmental effects for the two traits were assumed distributed as:

$$N\left(\left[\begin{array}{c}\mathbf{0}\\ \mathbf{0}\end{array}\right],\boldsymbol{ }\mathbf{I}\otimes \left[\begin{array}{cc}{\sigma }_{pe, T1}^{2}& {r}_{pe}{\sigma }_{pe,T1}{\sigma }_{pe,T2}\\ {r}_{pe}{\sigma }_{pe, T1}{\sigma }_{pe,T2}& {\sigma }_{pe,T2}^{2}\end{array}\right]\right),$$

where ${\sigma }_{pe, T1}^{2}$ (${\sigma }_{pe,T2}^{2}$) is the permanent environmental variance of trait 1 (trait 2) and ${r}_{pe}$ is the permanent environmental correlation between traits 1 and 2.

The above models assume genetic variance was the same for the four genetic groups. To evaluate possible differences in genetic variances between genetic groups, we also estimated heritabilities with the multivariate equivalent model of the previously described univariate model, with each trait modelled as three traits: one for WW, one for YY, and one for the crossbreds (WY and YW). In this multivariate model, residual and permanent environmental covariances between the three genetic groups were set to zero, because each individual only has phenotypes pertaining to one of the groups. Genetic covariances between the groups were also fixed to zero, to avoid that the estimate of genetic variance for a group was affected by information from the other genetic groups. Weighted averages of all variance components were estimated, using the proportion of animals in a genetic group as weights, i.e., 0.22 for WW, 0.25 for YY, and 0.53 for crossbreds. Heritability (${h}^{2}$) and repeatability (*r*) were estimated based on the weighted averages.

The predicted mean phenotypes of each genetic group based on the univariate model, i.e., the sum of the overall mean and the estimated effect of the genetic group ($\widehat{\mu }+\widehat{b}$), was obtained using the “predict” statement in ASReml 4.2.

Percent heterosis was estimated from these predicted means as:

$$Percent\,heterosis\,\left(H{\%}\right)=\frac{mean\,of\,the\,WY\,\left(YW\right)line-parental\,mean}{parental\,mean}\times 100\mathrm{\%},$$

where $parental\,mean$ is the average of the predicted means of the parental breeds. The reciprocal cross differences were estimated from these predicted values as:

$$Reciprocal\,cross\,differences\,\left({\%}\right)=\frac{mean\,of\,the\,WY\,line-mean\,of\,the\,YW\,line}{parental\,mean}\times 100{\%}.$$

Estimated differences between the predicted mean of YW and the parental mean, between the predicted mean of WY and the parental mean, and between the predicted means of YW and WY, as well as the Wald F statistics for these contrasts, were obtained using the “!CONTRAST” qualifier in ASReml 4.2. Because average pause length was not normally distributed, which might affect *p*-values, the Wald F statistics for this trait was carried out based on Box-Cox transformed data [27]. This transformation was performed using the “MASS” R-package (https://www.r-project.org/). Given the large number of traits evaluated, the p-values to declare significance were adjusted for multiple testing by computing false discovery rates (FDR) based on the Benjamini and Hochberg method [28] using the p.adjust function of the R software. This was done separately for each crossbred group. For each crossbred group, the number of comparisons is 70, since the deviation of the predicted mean from the parental mean was evaluated for 70 traits.

## Results

### Descriptive statistics

Descriptive statistics for egg weight, egg production, and egg quality till 100 weeks of age are in Table 1. Egg weight increased from 43.4 g for the first egg to 62.3 g at 100 weeks of age. The average age at first egg was 167.7 days. The chickens produced 5.8 eggs per week from 24 till 43 weeks of age, 4.6 eggs per week from 43 till 72 weeks of age, and 3.4 eggs per week from 72 till 100 weeks of age. We observed a decrease for average clutch length, from 7.6 to 4.2 days, an increase for number of clutches from 18.5 to 90.3, and an increase for average pause length from 1.3 to 2.1 days. Eggshell colour became darker, eggshell strength, eggshell thickness, eggshell ratio, and egg shape index decreased, while yolk ratio increased during the laying period. Haugh unit values were highest at 54 weeks of age.

**Table 1:** Descriptive statistics of egg-laying performance in purebred Beijing-You chickens, purebred White Leghorns and their reciprocal crosses

Trait	Abbreviation	NA	NO	Mean	SD	CV (%)
*Egg weight (g)*						
First three eggs	FEWt	1186	3485	43.35	4.81	11.9
28 weeks of age	EWt28	1142	3320	51.33	5.08	9.9
32 weeks of age	EWt32	1075	2977	53.16	4.84	9.1
36 weeks of age	EWt36	1025	3033	54.62	5.00	9.2
40 weeks of age	EWt40	990	2654	56.18	5.03	9.0
44 weeks of age	EWt44	1000	4708	56.76	5.01	8.8
48 weeks of age	EWt48	897	2569	58.03	5.12	8.8
52 weeks of age	EWt52	869	2517	58.14	4.99	8.6
56 weeks of age	EWt56	852	2314	59.19	5.06	8.6
60 weeks of age	EWt60	825	1928	60.86	5.11	8.4
64 weeks of age	EWt64	835	1931	60.96	5.08	8.3
68 weeks of age	EWt68	823	4080	60.98	5.22	8.6
72 weeks of age	EWt72	734	2022	60.35	5.22	8.7
76 weeks of age	EWt76	598	1230	61.47	5.38	8.8
86 weeks of age	EWt86	640	1793	61.06	5.69	9.3
100 weeks of age	EWt100	578	1689	62.25	5.90	9.5
*Egg production*						
Age at first egg (day of age)	AFE	1116	1116	167.72	11.86	7.1
Oviposition period (hours)	OP	1103	1103	25.27	1.11	4.4
*Egg production till 43 weeks of age*						
Cumulative egg number	EN43	936	936	110.87	18.52	16.7
Number of clutches	NC43	961	961	18.54	7.92	42.7
Average clutch length (days)	ACL43	948	948	7.56	6.09	80.6
Average pause length (days)	APL43	939	939	1.25	0.44	34.7
*Egg production till 72 weeks of age*						
Cumulative egg number	EN72	735	735	245.51	44.01	17.9
Number of clutches	NC72	752	752	53.11	20.23	38.1
Average clutch length (days)	ACL72	748	748	5.46	3.55	65.0
Average pause length (days)	APL72	751	751	1.61	1.58	97.8
*Egg production till 100 weeks of age*						
Cumulative egg number	EN100	702	702	340.94	76.78	22.5
Number of clutches	NC100	714	714	90.28	31.28	34.7
Average clutch length (days)	ACL100	712	712	4.19	2.16	51.6
Average pause length (days)	APL100	712	712	2.14	2.28	106.4
*Egg quality at 32 weeks of age*						
Egg shape index	ESI32	1078	3152	76.42	2.93	3.8
Eggshell colour (%)	ESC32	1077	3168	64.62	13.21	20.5
Eggshell strength (kg/cm^2^)	ESS32	1072	3123	3.80	0.67	17.8
Eggshell thickness (mm)	EST32	1076	3145	0.34	0.03	8.1
Eggshell ratio	ESR32	1071	2957	9.98	0.71	7.1
Yolk ratio	YR32	1074	2883	29.07	2.47	8.5
Yolk colour	YC32	1075	3023	5.91	1.29	21.9
Haugh unit	HU32	1074	2952	66.88	9.57	14.3
*Egg quality at 54 weeks of age*						
Egg shape index	ESI54	856	2380	73.63	2.93	4.0
Eggshell colour (%)	ESC54	855	2408	66.31	11.53	17.4
Eggshell strength (kg/cm^2^)	ESS54	855	2395	3.54	0.74	20.9
Eggshell thickness (mm)	EST54	854	2405	0.34	0.03	8.2
Eggshell ratio	ESR54	854	2377	9.30	0.77	8.3
Yolk ratio	YR54	855	2350	30.45	2.32	7.6
Yolk colour	YC54	855	2383	4.70	0.86	18.3
Haugh unit	HU54	856	2377	75.54	6.91	9.1
*Egg quality at 72 weeks of age*						
Egg shape index	ESI72	734	2047	73.22	3.28	4.5
Eggshell colour (%)	ESC72	733	2061	61.52	11.15	18.1
Eggshell strength (kg/cm^2^)	ESS72	732	2043	3.42	0.80	23.5
Eggshell thickness (mm)	EST72	733	2051	0.32	0.03	10.1
Eggshell ratio	ESR72	732	1990	9.09	1.06	11.6
Yolk ratio	YR72	734	1977	30.70	2.54	8.3
Yolk colour	YC72	735	2030	6.76	1.38	20.4
Haugh unit	HU72	733	1990	73.41	9.60	13.1
*Egg quality at 86 weeks of age*						
Egg shape index	ESI86	645	1831	72.76	3.55	4.9
Eggshell colour (%)	ESC86	644	1829	59.60	9.97	16.7
Eggshell strength (kg/cm^2^)	ESS86	644	1816	3.20	0.91	28.3
Eggshell thickness (mm)	EST86	632	1585	0.34	0.03	9.9
Eggshell ratio	ESR86	628	1527	8.75	0.98	11.1
Yolk ratio	YR86	638	1739	30.94	2.48	8.0
Yolk colour	YC86	636	1616	6.33	1.61	25.5
Haugh unit	HU86	642	1760	67.98	11.95	17.6
*Egg quality at 100 weeks of age*						
Egg shape index	ESI100	580	1674	72.80	3.82	5.2
Eggshell colour (%)	ESC100	576	1686	57.30	9.76	17.0
Eggshell strength (kg/cm^2^)	ESS100	581	1656	2.96	0.83	28.1
Eggshell thickness (mm)	EST100	580	1658	0.30	0.04	15.0
Eggshell ratio	ESR100	576	1620	8.03	1.21	15.1
Yolk ratio	YR100	567	1550	30.31	2.69	8.9
Yolk colour	YC100	574	1597	5.81	1.65	28.4
Haugh unit	HU100	574	1581	65.51	13.14	20.1

The descriptive statistics were computed across all animals

*NA* number of animals, *NO* number of observations, *SD* standard deviation, *CV* coefficient of variance

### Heritabilities and correlations

Estimates of heritabilities, repeatabilities, and phenotypic variance for egg weight traits are in Table 2. With the univariate model, estimated heritabilities for egg weights were high (0.41–0.75), except for the first egg weight (0.29). Repeatabilities showed a similar trend as heritabilities, with the lowest repeatability estimate for the first egg weight. With the multivariate model, estimated heritabilities for egg weights ranged from 0.28 to 0.73, and repeatabilities ranged from 0.47 to 0.85. Estimates of genetic and phenotypic correlations for egg weight traits are in Table 3. Estimates of genetic correlations between egg weights at different ages were high (0.72–1.00), especially between consecutive time points. The phenotypic correlation between egg weights at consecutive time points was larger than 0.41.

**Table 2:** Estimates of variances, heritabilities, and repeatabilities for egg weight traits at different ages

Trait	Univariate model			Multivariate model		
	Phenotypic variance (${\sigma }_{p}^{2}$)	Heritability (*h*^*2*^)	Repeatability (*r*)	Phenotypic variance (${\sigma }_{p}^{2}$)	Heritability (*h*^*2*^)	Repeatability (*r*)
FEWt	19.87	0.29	0.47	19.94	0.28	0.47
EWt28	13.02	0.46	0.71	12.99	0.46	0.71
EWt32	13.43	0.51	0.70	13.35	0.49	0.69
EWt36	14.74	0.62	0.85	14.59	0.63	0.85
EWt40	15.97	0.62	0.85	15.97	0.62	0.85
EWt44	16.27	0.58	0.77	16.13	0.58	0.77
EWt48	18.41	0.75	0.85	17.64	0.73	0.84
EWt52	18.40	0.69	0.81	17.89	0.68	0.81
EWt56	19.49	0.64	0.83	19.08	0.61	0.83
EWt60	19.69	0.52	0.84	19.73	0.54	0.84
EWt64	20.67	0.68	0.85	20.38	0.71	0.85
EWt68	21.80	0.57	0.79	21.60	0.58	0.79
EWt72	21.91	0.45	0.70	21.58	0.41	0.69
EWt76	23.34	0.55	0.80	22.55	0.53	0.80
EWt86	28.17	0.56	0.74	27.72	0.49	0.74
EWt100	30.01	0.41	0.74	29.31	0.46	0.74

With the univariate model, standard errors ranged from 0.05 to 0.11 for heritabilities, and from 0.01 to 0.02 for repeatabilities. With the multivariate model, standard errors ranged from 0.05 to 0.11 for heritabilities, and from 0.01 to 0.02 for repeatabilities

*FEWt* average weight for the first three eggs, *EWtX* egg weight at X weeks of age

**Table 3:** Estimates of genetic and phenotypic correlations for egg weight traits at different ages (in weeks)

Trait	FEWt	EWt28	EWt32	EWt36	EWt40	EWt44	EWt48	EWt52	EWt56	EWt60	EWt64	EWt68	EWt72	EWt76	EWt86	EWt100
FEWt		0.74	0.83	0.75	0.77	0.77	0.78	0.68	0.76	0.78	0.79	0.80	0.75	0.80	0.76	0.72
EWt28	0.41		0.95	0.92	0.90	0.87	0.85	0.84	0.83	0.83	0.88	0.85	0.82	0.83	0.76	0.82
EWt32	0.48	0.61		1.00	1.00	0.94	0.95	0.93	0.89	0.91	0.97	0.96	0.94	0.96	0.86	0.92
EWt36	0.44	0.62	0.71		0.98	0.94	0.93	0.92	0.91	0.89	0.94	0.94	0.92	0.93	0.83	0.93
EWt40	0.44	0.59	0.69	0.77		1.00	0.97	0.96	0.95	0.93	0.98	0.96	0.96	0.99	0.92	0.97
EWt44	0.39	0.53	0.62	0.70	0.73		0.99	0.98	0.98	0.97	0.98	0.99	0.96	0.99	0.92	0.97
EWt48	0.40	0.55	0.64	0.72	0.76	0.75		0.99	1.00	1.00	1.00	0.99	0.99	0.99	0.94	0.98
EWt52	0.36	0.53	0.61	0.68	0.72	0.71	0.76		0.99	0.99	0.99	0.98	0.97	0.99	0.92	0.99
EWt56	0.35	0.53	0.62	0.68	0.72	0.70	0.76	0.77		0.99	1.00	1.00	0.98	0.99	0.97	1.00
EWt60	0.37	0.52	0.60	0.64	0.68	0.66	0.69	0.71	0.72		0.99	0.97	1.00	0.99	0.96	1.00
EWt64	0.38	0.52	0.59	0.67	0.69	0.67	0.72	0.73	0.75	0.72		0.99	1.00	1.00	0.96	0.98
EWt68	0.35	0.49	0.56	0.64	0.64	0.64	0.70	0.69	0.72	0.69	0.76		1.00	1.00	0.98	0.98
EWt72	0.31	0.45	0.51	0.57	0.59	0.56	0.62	0.62	0.65	0.62	0.70	0.70		0.99	0.99	0.97
EWt76	0.36	0.53	0.57	0.61	0.63	0.58	0.66	0.64	0.65	0.63	0.72	0.71	0.67		0.99	0.98
EWt86	0.36	0.44	0.52	0.54	0.56	0.52	0.60	0.58	0.62	0.56	0.64	0.61	0.64	0.72		0.98
EWt100	0.29	0.40	0.47	0.49	0.47	0.47	0.54	0.53	0.53	0.55	0.56	0.55	0.54	0.57	0.60	

Genetic correlations are above the diagonal and phenotypic correlations are below the diagonal. Standard errors ranged from 0.01 to 0.12 for genetic correlations, and from 0.01 to 0.03 for phenotypic correlations

*FEWt* weight for the first three egg, *EWtX* egg weight at X weeks of age

Estimates of heritabilities and phenotypic variance for egg production traits are in Table 4. With the univariate model, relatively high heritabilities were estimated for age at first egg (0.55), oviposition period (0.38), and number of clutches (0.49, 0.40, and 0.27 at 43, 72, and 100 weeks of age). With the multivariate model, age at first egg, oviposition period, and number of clutches also showed relatively high heritability estimates. Cumulative egg number till 43, 72, and 100 weeks of age showed low heritability estimates. Estimates of genetic and phenotypic correlations for egg production traits are in Table 5. EN43 was estimated to be highly genetically correlated with age at first egg (r_g_ = − 0.70) and EN72 (r_g_ = 0.46), and moderately with average clutch length at 43 weeks of age (r_g_ = 0.29) and oviposition period (r_g_ = − 0.36). EN72 was estimated to be negatively genetically correlated with oviposition period (r_g_ = − 0.40), and number of clutches at 72 weeks of age (r_g_ = − 0.54). EN100 was positively correlated with EN72 (r_g_ = 0.90), with average clutch length at 43 weeks of age (r_g_ = 0.69), and with average clutch length at 72 weeks of age (r_g_ = 0.57). Bivariate models that included average pause length at 43, 72 or 100 weeks of age did not converge.

**Table 4:** Estimates of variances and heritabilities for egg production traits

Trait	Univariate model		Multivariate model	
	Phenotypic variance (${\sigma }_{p}^{2}$)	Heritability (*h*^*2*^)	Phenotypic variance (${\sigma }_{p}^{2}$)	Heritability (*h*^*2*^)
AFE	81.18	0.55	82.78	0.62
OP	0.78	0.38	0.82	0.47
EN43	173.21	0.15	171.54	0.16
NC43	45.54	0.49	44.88	0.46
ACL43	25.88	0.05	23.76	0.29
APL43	0.19	0.01	0.19	0.04
EN72	1184.40	0.14	1152.90	0.10
NC72	237.93	0.40	240.91	0.41
ACL72	7.40	0.08	6.92	0.28
APL72	2.38	0.01	2.39	0.01
EN100	4309.40	0.15	4188.30	0.12
NC100	667.33	0.27	675.45	0.35
ACL100	2.62	0.11	2.49	0.30
APL100	5.21	0.13	5.12	0.18

Standard errors for heritabilities ranged from 0.02 to 0.09 for the univariate model, and from 0.02 to 0.14 for the multivariate model

*AFE* age at first egg, *OP* oviposition period, *ENX* cumulative egg number till X weeks of age, *NCX* number of clutches till X weeks of age, *ACLX* average clutch length till X weeks of age, *APLX* average pause length till X weeks of age

**Table 5:** Estimates of genetic and phenotypic correlations for egg production traits

Trait	AFE	OP	EN43	NC43	ACL43	APL43	EN72	NC72		ACL72	APL72	EN100	NC100	ACL100	APL100
AFE		− 0.26	− 0.70	− 0.57	0.29	DNC	0.01	− 0.43		0.28	DNC	0.27	− 0.31	0.21	DNC
OP	− 0.001		− 0.36	0.87	− 0.69	DNC	− 0.40	0.87		− 0.62	DNC	− 0.29	0.71	− 0.59	DNC
EN43	− 0.47	− 0.36		− 0.23	0.29	DNC	0.46	− 0.24		0.18	DNC	0.18	− 0.28	0.35	DNC
NC43	− 0.26	0.71	− 0.40		− 0.61	DNC	− 0.50	0.98		− 0.88	DNC	− 0.42	0.83	− 0.80	DNC
ACL43	0.05	− 0.38	0.42	− 0.82		DNC	0.73	− 0.82		0.98	DNC	0.69	− 0.67	0.99	DNC
APL43	DNC	DNC	DNC	DNC	DNC		DNC	DNC		DNC	DNC	DNC	DNC	DNC	DNC
EN72	− 0.17	− 0.30	0.70	− 0.45	0.47	DNC		− 0.54		0.63	DNC	0.90	− 0.33	0.68	DNC
NC72	− 0.13	0.57	− 0.28	0.78	− 0.50	DNC	− 0.25			− 0.63	DNC	− 0.44	0.91	− 0.83	DNC
ACL72	0.02	− 0.37	0.41	− 0.58	0.81	DNC	0.53	− 0.89			DNC	0.57	− 0.83	0.99	DNC
APL72	DNC	DNC	DNC	DNC	DNC	DNC	DNC	DNC		DNC		DNC	DNC	DNC	DNC
EN100	− 0.08	− 0.18	0.55	− 0.33	0.41	DNC	0.87	− 0.20		0.49	DNC		− 0.05	0.20	DNC
NC100	− 0.08	0.44	− 0.07	0.55	− 0.29	DNC	− 0.01	0.84		− 0.43	DNC	0.19		− 0.56	DNC
ACL100	0.01	− 0.39	0.37	− 0.54	0.64	DNC	0.52	− 0.67		0.87	DNC	0.11	− 0.79		DNC
APL100	DNC	DNC	DNC	DNC	DNC	DNC	DNC	DNC		DNC	DNC	DNC	DNC	DNC	

Genetic correlations are above the diagonal and phenotypic correlations are below the diagonal. Standard errors ranged from 0.02 to 0.46 for genetic correlations, and from 0.01 to 0.05 for phenotypic correlations

*AFE* age at first egg, *OP* oviposition period, *ENX* cumulative egg number till X weeks of age, *NCX* number of clutches till X weeks of age, *ACLX* average clutch length till X weeks of age, *APLX* average pause length till X weeks of age, *DNC* did not converge

Estimates of heritabilities, repeatabilities, and phenotypic variance for egg quality traits at 32, 54, 72, 86, and 100 weeks of age are in Table 6. With the univariate model, heritability estimates were low for egg shape index (0.18–0.32), yolk colour (0.12–0.22), and Haugh unit (0.16–0.29). Heritability estimates for eggshell colour, eggshell strength, eggshell thickness, eggshell ratio, and yolk ratio decreased during the laying period. Repeatability estimates were low for eggshell thickness and eggshell ratio. With the multivariate model, heritability estimates were low for egg shape index, eggshell strength, eggshell thickness, and yolk colour. Estimates of genetic and phenotypic correlations for egg quality traits at 32, 54, 72, 86, and 100 weeks of age are in Table 7. Eggshell strength had a strong genetic correlation estimate with eggshell thickness (0.43–0.85) and eggshell ratio (0.45–0.98). Estimates of phenotypic correlations between eggshell strength, eggshell thickness, and eggshell ratio were similar and high at 32, 54, 72, 86, and 100 weeks of age. Phenotypic correlation estimates among other egg quality traits were different and low across different ages. Estimates of genetic correlations for the same egg quality trait measured at different ages were high, ranging from 0.57 to 1.00 (see Additional file 2: Fig. S1).

**Table 6:** Estimates of variances, heritabilities, and repeatabilities for egg quality traits at different ages

Model	Trait	32 weeks of age			54 weeks of age			72 weeks of age			86 weeks of age			100 weeks of age		
		${\sigma }_{p}^{2}$	*h*^*2*^	*r*	${\sigma }_{p}^{2}$	*h*^*2*^	*r*	${\sigma }_{p}^{2}$	*h*^*2*^	*r*	${\sigma }_{p}^{2}$	*h*^*2*^	*r*	${\sigma }_{p}^{2}$	*h*^*2*^	*r*
Univariate model	ESI	7.42	0.32	0.41	8.01	0.19	0.43	11.06	0.18	0.42	12.92	0.29	0.50	15.20	0.27	0.47
	ESC	42.94	0.37	0.65	34.00	0.32	0.58	36.21	0.39	0.60	30.46	0.24	0.68	28.23	0.19	0.68
	ESS	0.38	0.26	0.51	0.48	0.18	0.48	0.59	0.14	0.48	0.80	0.22	0.48	0.70	0.12	0.49
	EST	7.23E-04	0.25	0.47	6.98E−04	0.20	0.39	9.58E−04	0.03	0.40	1.06E−03	0.18	0.36	1.91E−03	0.03	0.18
	ESR	0.47	0.33	0.56	0.59	0.27	0.50	1.10	0.07	0.32	0.93	0.19	0.39	1.48	0.08	0.22
	YR	4.09	0.29	0.51	3.67	0.46	0.74	4.72	0.21	0.56	4.95	0.17	0.66	6.29	0.22	0.58
	YC	1.66	0.12	0.47	0.68	0.22	0.47	1.69	0.18	0.41	2.56	0.18	0.62	2.76	0.19	0.62
	HU	91.22	0.16	0.18	47.12	0.29	0.57	91.35	0.20	0.42	142.30	0.27	0.40	174.29	0.23	0.53
Multivariate model	ESI	7.53	0.27	0.42	8.49	0.24	0.47	10.97	0.14	0.42	12.83	0.26	0.50	15.21	0.24	0.48
	ESC	43.86	0.44	0.65	35.98	0.42	0.60	36.35	0.41	0.60	31.01	0.24	0.70	30.22	0.26	0.71
	ESS	0.39	0.26	0.51	0.48	0.17	0.47	0.60	0.17	0.48	0.80	0.19	0.48	0.70	0.13	0.50
	EST	7.22E-04	0.25	0.47	7.24E−04	0.23	0.41	9.75E−04	0.13	0.42	1.05E−03	0.16	0.36	1.93E−03	0.07	0.18
	ESR	0.47	0.31	0.56	0.58	0.27	0.48	1.11	0.10	0.32	0.94	0.18	0.38	1.49	0.11	0.23
	YR	4.32	0.36	0.52	3.73	0.45	0.74	4.86	0.28	0.58	4.89	0.16	0.65	6.28	0.27	0.58
	YC	1.61	0.12	0.44	0.69	0.18	0.48	1.77	0.18	0.43	2.58	0.21	0.62	2.74	0.20	0.61
	HU	90.43	0.14	0.18	46.13	0.34	0.56	90.16	0.22	0.41	139.69	0.26	0.39	171.70	0.23	0.51

With the univariate model, standard errors ranged from 0.04 to 0.09 for heritabilities, and from 0.02 to 0.03 for repeatabilities. With the multivariate model, standard errors ranged from 0.03 to 0.11 for heritabilities, and from 0.01 to 0.03 for repeatabilities

*ESI* egg shape index, *ESC* eggshell colour, *ESS* eggshell strength, *EST* eggshell thickness, *ESR* eggshell ratio, *YR* yolk ratio, *YC* yolk colour, *HU* Haugh unit, ${\sigma }_{p}^{2}$ phenotypic variance, *h*^*2*^ heritability estimate with standard errors in parentheses, *r* repeatability estimate with standard errors in parentheses

**Table 7:** Estimates of genetic and phenotypic correlations for egg quality traits at different ages

Trait	ESI	ESC	ESS	EST	ESR	YR	YC	HU
ESI32		0.01	− 0.04	0.02	0.08	0.03	− 0.10	0.08
ESC32	0.02		− 0.20	− 0.40	− 0.37	0.22	0.03	− 0.51
ESS32	0.05	− 0.14		0.67	0.76	− 0.01	0.28	− 0.10
EST32	− 0.02	− 0.31	0.43		0.83	− 0.10	0.34	− 0.24
ESR32	0.03	− 0.19	0.54	0.64		0.11	0.32	− 0.18
YR32	0.02	0.11	0.02	− 0.09	0.12		0.13	− 0.33
YC32	− 0.02	0.03	0.01	− 0.02	0.03	0.06		− 0.04
HU32	0.16	0.05	0.01	− 0.06	− 0.09	− 0.17	− 0.07	
ESI54		0.00	0.00	0.05	− 0.11	0.22	− 0.31	0.13
ESC54	0.01		− 0.30	− 0.30	− 0.25	− 0.01	− 0.13	− 0.11
ESS54	0.09	− 0.11		0.70	0.70	0.09	0.23	0.28
EST54	0.10	− 0.09	0.49		0.89	− 0.02	0.03	0.10
ESR54	0.09	− 0.16	0.51	0.71		0.05	0.07	− 0.11
YR54	0.07	0.05	− 0.02	− 0.05	− 0.01		− 0.06	− 0.20
YC54	− 0.06	0.00	− 0.01	− 0.05	− 0.03	0.07		− 0.15
HU54	− 0.01	− 0.09	0.01	− 0.09	− 0.07	− 0.22	− 0.03	
ESI72		− 0.25	− 0.02	− 0.30	0.04	0.22	− 0.36	0.48
ESC72	− 0.04		− 0.24	− 0.46	− 0.33	− 0.29	0.39	− 0.07
ESS72	0.03	− 0.15		0.43	0.58	0.38	− 0.32	0.05
EST72	0.04	− 0.11	0.46		0.81	0.06	0.19	− 0.46
ESR72	0.03	− 0.16	0.39	0.57		0.16	− 0.04	− 0.34
YR72	0.07	− 0.02	0.01	− 0.07	0.07		0.13	0.18
YC72	− 0.03	0.03	0.02	− 0.01	− 0.03	0.09		− 0.33
HU72	0.05	− 0.06	0.04	− 0.04	0.00	− 0.09	− 0.13	
ESI86		0.14	− 0.33	− 0.37	− 0.63	0.09	− 0.34	0.12
ESC86	0.05		− 0.09	− 0.05	0.02	0.02	− 0.07	− 0.28
ESS86	− 0.04	− 0.15		0.44	0.45	0.24	0.41	0.59
EST86	− 0.03	− 0.14	0.48		DNC	0.01	0.16	− 0.08
ESR86	− 0.04	− 0.10	0.46	DNC		0.07	0.18	− 0.11
YR86	− 0.02	− 0.03	− 0.07	− 0.18	0.01		0.03	− 0.12
YC86	− 0.10	− 0.04	− 0.05	− 0.08	− 0.05	0.16		0.21
HU86	0.05	− 0.04	0.08	− 0.06	0.03	− 0.04	0.05	
ESI100		− 0.22	− 0.56	− 0.89	− 0.83	0.20	− 0.08	0.24
ESC100	− 0.01		− 0.20	− 0.12	− 0.09	− 0.22	0.04	0.16
ESS100	− 0.03	− 0.11		0.85	0.98	0.34	− 0.10	0.05
EST100	− 0.01	− 0.12	0.43		DNC	0.93	0.31	DNC
ESR100	0.02	− 0.06	0.43	DNC		0.27	0.22	− 0.28
YR100	0.03	0.00	− 0.04	− 0.10	− 0.05		0.17	− 0.28
YC100	− 0.08	0.07	0.01	− 0.02	− 0.01	0.14		− 0.02
HU100	0.04	− 0.05	0.04	DNC	0.01	− 0.12	− 0.01	

Genetic correlations are above the diagonal and phenotypic correlations are below the diagonal. Standard errors ranged from 0.04 to 0.64 for genetic correlations, and from 0.01 to 0.04 for phenotypic correlations

*ESIX* egg shape index at X weeks of age, *ESCX* eggshell colour at X weeks of age, *ESSX* eggshell strength at X weeks of age, *ESTX* eggshell thickness at X weeks of age, *ESRX* eggshell ratio at X weeks of age, *YRX* yolk ratio at X weeks of age, *YCX* yolk colour at X weeks of age, *HUX* Haugh unit at X weeks of age, *DNC* did not converge

### Heterosis and reciprocal cross differences for egg-laying performance

Predicted averages and estimates of heterosis for egg weight are in Table 8. Both crossbreds showed significant heterosis for egg weight, except for first egg weight, ranging from 1.9 to 9.0% (*FDR* ≤ 0.01). Heterosis for egg weight increased with age. The egg weights of YW and WY were higher than the best parental breed, WW, from 56 and 76 weeks of age, respectively. Estimates of reciprocal cross differences were not significant for egg weights measured along the laying period, ranging from − 4.1 to − 1.5% (*FDR* > 0.05).

**Table 8:** Predicted egg weight for the four genetic groups and estimates of heterosis for the two reciprocal crosses

Trait	Genetic group				H% (WY)	H% (YW)	Reciprocal cross differences (%)
	WW	YY	WY	YW			
FEWt	44.97	40.55	43.20	44.95	1.0	5.1**	− 4.1
EWt28	55.63	45.52	51.53	52.85	1.9**	4.5**	− 2.6
EWt32	57.01	48.06	53.84	54.61	2.5**	4.0**	− 1.5
EWt36	58.25	49.33	54.96	56.15	2.2**	4.4**	− 2.2
EWt40	59.34	50.52	56.39	57.42	2.7**	4.5**	− 1.9
EWt44	59.80	51.47	56.98	58.26	2.4**	4.7**	− 2.3
EWt48	60.74	52.87	58.35	59.75	2.7**	5.2**	− 2.5
EWt52	60.39	53.21	58.37	60.04	2.8**	5.7**	− 2.9
EWt56	60.81	54.58	59.30	61.26	2.8**	6.2**	− 3.4
EWt60	62.41	55.90	60.90	62.55	3.0**	5.7**	− 2.8
EWt64	62.23	56.26	61.18	62.81	3.3**	6.0**	− 2.8
EWt68	62.01	56.34	61.42	62.98	3.8**	6.4**	− 2.6
EWt72	61.24	55.92	60.95	62.52	4.0**	6.7**	− 2.7
EWt76	62.17	56.73	62.33	63.41	4.8**	6.7**	− 1.8
EWt86	60.87	56.93	62.25	63.17	5.7**	7.2**	− 1.6
EWt100	61.85	57.82	62.80	65.20	4.9**	9.0**	− 4.0

H% (WY): percent heterosis for WY, the percentage of performance of WY being better than the average performance of the two parental lines, H% (YW): percent heterosis for YW, the percentage of performance of YW being better than the average performance of the two parental lines

Wald F statistics after adjusting with multiple testing for H% (WY), H% (YW), and reciprocal cross differences are indicated as follows *FDR ≤ 0.05, **FDR ≤ 0.01

*YY* Beijing-You chickens, *WW* White Leghorn chickens, *WY* offspring of a cross between White Leghorn as the sire line and Beijing-You as the dam line, *YW* offspring of a cross between Beijing-You as the sire line and White Leghorn as the dam line, *FEWt* weight for the first three eggs, *EWtX* egg weight at X weeks of age

Predicted averages and estimates of heterosis for egg production traits are in Table 9. Age at first egg, EN72, and EN100 showed significant and favourable heterosis (*FDR* ≤ 0.05). The estimate of heterosis for oviposition period was not significant (*FDR* > 0.05). Average clutch length till 43, 72, and 100 weeks of age showed high and negative heterosis (*FDR* ≤ 0.01). Favourable and significant heterosis for average pause length was observed for WY at 72 weeks of age (*FDR* ≤ 0.05), and for both crossbreds at 100 weeks of age (*FDR* ≤ 0.01). The difference between the reciprocal crosses was not significant for any egg production trait (*FDR* > 0.05).

**Table 9:** Predicted egg production related traits for the four genetic groups and estimates of heterosis for the two reciprocal crosses

Trait	Genetic group				H% (WY)	H% (YW)	Reciprocal cross differences (%)
	WW	YY	WY	YW			
AFE	157.69	180.70	165.80	167.02	− 2.0**	− 1.3*	− 0.7
OP	24.31	26.21	25.11	25.29	− 0.6	0.1	− 0.7
EN43	129.03	92.06	113.29	112.14	2.5*	1.4	1.0
NC43	11.76	23.62	18.16	18.67	2.7	5.6	− 2.9
ACL43	14.16	3.95	6.79	6.45	− 25.0**	− 28.8**	3.8
APL43	1.30	1.26	1.24	1.22	− 3.1	− 4.9	1.7
EN72	280.65	201.23	253.34	247.39	5.1**	2.7*	2.5
NC72	33.18	70.86	53.06	51.31	2.0	− 1.4	3.4
ACL72	9.72	2.89	4.98	4.90	− 21.1**	− 22.2**	1.1
APL72	1.59	1.88	1.47	1.52	− 15.3*	− 12.6	− 2.7
EN100	379.91	270.33	362.67	352.77	11.6**	8.5**	3.0
NC100	59.83	110.21	94.83	90.23	11.5**	6.1*	5.4
ACL100	6.75	2.50	3.97	3.93	− 14.2**	− 15.1**	0.9
APL100	2.57	2.41	1.88	1.93	− 24.6**	− 22.3**	− 2.3

H% (WY): percent heterosis for WY, the percentage of performance of WY being better than the average performance of the two parental lines, H% (YW): percent heterosis for YW, the percentage of performance of YW being better than the average performance of the two parental lines

Wald F statistics after adjusting with multiple testing for H% (WY), H% (YW), and reciprocal cross differences are indicated as follows *FDR ≤ 0.05, **FDR ≤ 0.01

*YY* Beijing-You chickens, *WW* White Leghorn chickens, *WY* offspring of a cross between White Leghorn as the sire line and Beijing-You as the dam line, *YW* offspring of a cross between Beijing-You as the sire line and White Leghorn as the dam line, *AFE* age at first egg, *OP* oviposition period, *ENX* cumulative egg number till X weeks of age, *NCX* number of clutches till X weeks of age, *ACLX* average clutch length till X weeks of age, *APLX* average pause length till X weeks of age

Predicted averages and heterosis for egg quality traits are in Table 10. All egg quality traits showed positive heterosis, except for egg shape index, yolk ratio, yolk colour, and Haugh unit. At the investigated time points, significant heterosis was observed for eggshell colour and eggshell thickness for YW (*FDR* ≤ 0.01) and for eggshell colour and Haugh unit for WY (*FDR* ≤ 0.01). Eggshell strength of YW showed greater heterosis than other egg quality traits at early laying stages, 10.3, 12.6, and 11.4% at 32, 54, and 72 weeks of age, respectively. The difference between reciprocal crosses was significant for eggshell strength at 54 weeks of age (*FDR* ≤ 0.05) but not for other ages or other traits.

**Table 10:** Predicted egg quality traits for the four genetic groups and estimates of heterosis for the two reciprocal crosses

Trait	Genetic group				H% (WY)	H% (YW)	Reciprocal cross differences (%)
	WW	YY	WY	YW			
ESI32	75.09	78.02	76.04	76.21	− 0.7*	− 0.4	− 0.2
ESC32	79.25	46.68	68.11	66.49	8.2**	5.6**	2.6
ESS32	3.29	3.95	3.80	4.00	4.8**	10.3**	− 5.5
EST32	0.33	0.33	0.34	0.34	0.8	2.7**	− 1.9
ESR32	9.63	10.07	9.97	10.10	1.2	2.5**	− 1.3
YR32	26.56	30.64	29.16	29.22	1.9**	2.2**	− 0.2
YC32	5.68	6.06	6.14	5.78	4.6**	− 1.5	6.1
HU32	69.48	66.66	66.24	65.79	− 2.7**	− 3.3**	0.6
ESI54	73.36	74.28	73.42	73.36	− 0.5	− 0.6	0.1
ESC54	78.68	49.06	68.89	66.38	7.9**	3.9**	3.9
ESS54	3.08	3.75	3.44	3.84	0.9	12.6**	− 11.7*
EST54	0.33	0.33	0.33	0.35	0.6	4.7**	− 4.1
ESR54	9.00	9.36	9.22	9.45	0.5	2.9**	− 2.4
YR54	28.21	32.24	30.65	30.81	1.4*	1.9**	− 0.5
YC54	4.30	5.07	4.77	4.66	1.9	− 0.4	2.3
HU54	78.60	74.86	73.68	74.56	− 4.0**	− 2.8**	− 1.2
ESI72	72.79	73.60	73.18	73.13	− 0.01	− 0.1	0.1
ESC72	73.67	45.33	63.55	62.84	6.8**	5.6**	1.2
ESS72	2.92	3.63	3.39	3.65	3.6	11.4**	− 7.8
EST72	0.31	0.31	0.32	0.33	4.1**	6.6**	− 2.4
ESR72	8.80	9.06	9.16	9.28	2.6**	3.9**	− 1.3
YR72	28.61	32.70	30.64	30.88	− 0.04	0.7	− 0.8
YC72	6.31	7.29	6.72	6.76	− 1.2	− 0.6	− 0.6
HU72	76.25	73.76	70.78	72.90	− 5.6**	− 2.8	− 2.8
ESI86	72.23	72.62	72.72	72.89	0.4	0.6	− 0.2
ESC86	69.28	44.75	62.42	60.98	9.5**	7.0**	2.5
ESS86	2.78	3.47	3.19	3.32	2.2	6.3*	− 4.1
EST86	0.33	0.33	0.34	0.35	3.3**	5.9**	− 2.6
ESR86	8.50	8.74	8.81	8.93	2.2*	3.6**	− 1.4
YR86	29.07	32.64	30.76	31.21	− 0.3	1.1	− 1.4
YC86	5.97	6.95	6.13	6.34	− 5.1	− 1.8	− 3.3
HU86	70.53	69.49	63.91	66.51	− 8.7**	− 5.0**	− 3.7
ESI100	72.67	73.33	72.74	72.40	− 0.4	− 0.8	0.5
ESC100	66.24	41.33	58.95	57.37	9.6**	6.7**	2.9
ESS100	2.71	3.05	2.97	2.91	3.0	1.1	2.0
EST100	0.30	0.29	0.29	0.30	0.2	4.2**	− 4.0
ESR100	7.89	8.04	8.12	8.04	1.9	1.0	1.0
YR100	28.57	32.05	30.80	30.99	1.6	2.2*	− 0.6
YC100	5.17	6.41	5.84	6.15	0.7	6.1	− 5.4
HU100	68.16	67.66	61.25	64.49	− 9.8**	− 5.0**	− 4.8

H% (WY): percent heterosis for WY, the percentage of performance of WY being better than the average performance of the two parental lines, H% (YW): percent heterosis for YW, the percentage of performance of YW being better than the average performance of the two parental lines

Wald F statistics after adjusting with multiple testing for H% (WY), H% (YW), and reciprocal cross differences was indicated as follows *FDR ≤ 0.05, **FDR ≤ 0.01

*YY* Beijing-You chickens, *WW* White Leghorn chickens, *WY* offspring of a cross between White Leghorn as the sire line and Beijing-You as the dam line, *YW* offspring of a cross between Beijing-You as the sire line and White Leghorn as the dam line, *ESIX* egg shape index at X weeks of age, *ESCX* eggshell colour at X weeks of age, *ESSX* eggshell strength at X weeks of age, *ESTX* eggshell thickness at X weeks of age, *ESRX* eggshell ratio at X weeks of age, *YRX* yolk ratio at X weeks of age, *YCX* yolk colour at X weeks of age, *HUX* Haugh unit at X weeks of age

## Discussion

The objective of this study was to analyse the genetic background and estimate heterosis and the reciprocal cross differences for egg-laying performance for crosses between an indigenous chicken breed and a commercial layer breed throughout the laying period. It should be noted that the model that we used for analysis of crossbred performance is an alternative parameterization of the so-called Dickerson model that has been used previously in several other studies [29, 30]. The main difference is that heterosis and reciprocal effects are directly estimated in the Dickerson model, while they are obtained as differences between the estimated effects of the different genetic groups in our model. The equivalence between these two models is that the direct heterosis estimated by the Dickerson model is the same as the average heterosis (crossbred mean minus parental mean) in our model, and the reciprocal cross differences estimated by the Dickerson model is the same as the difference between the estimates of the crossbred means in our model (Additional file 3: Tables S4, S5, and S6). Conversely, our model directly estimates the average effects of the different genetic groups (as reported in Tables 8, 9, 10), while these can be obtained as a function of the estimated breed, heterosis and reciprocal effects in the Dickerson model.

### Genetic parameters

To relax the assumption of equal additive genetic variance for each of the genetic groups, we also calculated the genetic parameters with a multivariate model that allowed different genetic and residual variances for YY, WW, and crossbreds. Differences between estimates from the univariate and the multivariate model ranged from − 0.07 to 0.23 for heritabilities and from -0.03 to 0.03 for repeatabilities (see Additional file 4: Tables S7, S8, and S9). The difference in estimates of repeatability between the models was not significant (*p*-value = 0.16, as evaluated with the paired t-test). Although the differences in estimates of heritability between the models were significant (*p*-value = 0.006), the estimates were generally similar (see Additional file 5: Fig. S2, the correlation between the estimates of heritability from the two models was 0.914), with the average absolute difference being 0.037. For some traits, e.g., average clutch length at different timepoints, the estimated heritabilities were substantially different between the models. The high standard error of the estimate of heritability for average clutch length from the multivariate model (0.12–0.14) could be a possible explanation. In addition to estimating the weighted heritability and repeatability across all genetic groups, we also estimated the genetic parameters for YY, WW, and crossbreds, respectively (Additional file 6: Table S10, S11, and S12). Across all traits, WW tended to have lower heritabilities than YY and the crossbreds. This trend was most pronounced for egg weights, but was also generally the case for egg production and egg quality traits.

#### Egg weight

Overall, we obtained relatively high heritability estimates for egg weight, especially for egg weight at 48 weeks of age, which resulted from a high genetic variance and a low permanent environmental variance compared to other time points (Additional file 7: Table S13). These estimated heritabilities agreed with estimates from several other studies [31–33]. We obtained high repeatabilities for egg weight, suggesting that the variability in egg weights across weeks was relatively low. Furthermore, in accordance with previous studies [7, 34], strong genetic correlations were found for egg weights between time points, suggesting that the genetic determinants of egg weights are quite stable across ages. The exception of a relatively low genetic correlation estimate between the first egg weight and egg weight at later stages suggests a separate genetic background for weight of the first egg. This could be because the first eggs were collected at different ages across birds, which introduces other, age-related factors.

#### Egg production

The estimated heritabilities for EN43, EN72, and EN100 in the current study were low, similar to estimates in dual purpose chickens [32] and Rhode Island Red chickens [6], but lower than estimates for crossbred chickens (0.42 [35]). Another study has reported that crossbreds generally show a higher heritability than purebreds [31]. A stronger genetic correlation of oviposition period with EN72 (− 0.40) than with EN43 (− 0.36) and with EN100 (− 0.29) suggests that the oviposition period has a larger impact on egg number in the middle stage. This finding is similar to the results reported by Becot et al. [22], which confirmed that oviposition period showed a stronger genetic connection with laying rate at 44–64 weeks of age than that at 24–43 weeks of age.

#### Egg quality

All egg quality traits at the five evaluated time points showed low to moderate heritabilities in the current study. The estimated heritability decreased with age for eggshell strength, eggshell thickness, and eggshell ratio, which could be due to an increase of the residual variance (see Additional file 7: Table S14). A decrease in heritabilities with age for eggshell strength was also observed by Li et al. [36].

### Heterosis for egg-laying performance

In addition to genetic parameters, we also estimated heterosis and reciprocal cross differences with the multivariate model. The difference between the univariate model and the multivariate model ranged from − 1.04 to 1.24 in percentage points for heterosis of WY, from − 0.81 to 1.72 for heterosis of YW, and from − 1.58 to 1.52 for reciprocal cross differences (see Additional file 4: Tables S7, S8, and S9). On average across traits, the difference in estimates between models were not significantly different from zero for heterosis of WY, heterosis of YW, and the reciprocal cross differences (*p*-value = 0.65, *p*-value = 0.68, and *p*-value = 0.99, respectively based on a paired t-test). These additional results show that the assumption of equal genetic variance for all the genetic groups, as made in the univariate model, hardly affected estimates of heterosis for the traits evaluated here.

#### Egg weight

In the current study, positive heterosis was observed for egg weight and estimates were consistent with another study [19] that reported heterosis ranging from − 0.3 to 3.2% for crosses of different combinations of breeds. Based on a summary of studies conducted before 1990, it was concluded that heterosis for egg weight in different crosses ranged from − 3.0 to 5.0% [20]. Our estimates ranged from 1.0 to 9.0% at different time points, which agreed well with those previously reported estimates. Taken together, this suggests that egg weight has low to moderate heterosis, regardless of the parental line and age. It is widely accepted that heterosis is due to non-additive gene action [1]. Amuzu-Aweh et al. reported that the dominance variance could explain up to 3% of the phenotypic variance for egg weight [37]. The observed increase in heterosis with age in our study suggests that the non-additive effects may play a more important role at later laying stages.

#### Egg production

Egg number is an important economic trait for laying hens. In contrast to low and moderate heterosis for egg weights, estimates of heterosis for egg production have been highly variable in the literature, ranging from -3 to 40%, both in crosses between elite lines and in crosses between indigenous and elite lines [20]. We found low to moderate heterosis for crosses between the White Leghorn and Beijing-You, which both have a history of within-line selection. Some studies have shown that selection towards improved combining ability may have led to the accumulation of alleles that show heterosis and thus may exploit non-additive gene effects better than within-line selection [38, 39]. Differences in selection histories of the parental lines could be one of the reasons for differences in heterosis among studies.

We also found that the heterosis for cumulative egg number differed slightly across ages, i.e., 1.4 and 2.5% at 43 weeks of age for YW and WY, respectively, 2.7 and 5.1% at 72 weeks of age for YW and WY, respectively, and 8.5 and 11.6% at 100 weeks of age for YW and WY, respectively. The cumulative egg number was generated by summing up egg number over a period. Heterosis for egg number over four weeks showed fluctuations before 28 weeks of age (Additional file 8: Fig. S3), likely because of differences in age at first egg between birds, then decreased to nearly zero at the laying peak between 28 and 44 weeks of age, and increased thereafter. This trend was also shown by Ledur et al. in White Leghorn [40] and by Minvielle et al. in Japanese quails [41]. This trend is also consistent with the trend observed for heterosis for cumulative egg number, which showed fluctuations before 28 weeks of age, then a decrease between 28 and 44 weeks of age, and an increase thereafter (Additional file 8: Fig. S4). In addition, the crossbred chickens produced more eggs per week than the best parental line, WW, from 72 till 100 weeks of age, which implies that the crossbreds had better egg-laying persistency than the purebreds for the extended production period. A similar observation was reported in quails for cumulative egg number till 92 weeks of age [41].

Heterosis for egg number at each period was observed at the beginning and at the late egg-laying stage, but not at the laying peak. Egg number is a complex trait that is influenced by several components, including age at first egg, oviposition period, number of clutches, and average clutch (pause) length. Heterosis for egg number at the beginning of egg laying could be partly explained by significant and favourable heterosis for age at first egg. Heterosis for egg number at the late laying stage can be explained by the high and favourable heterosis for average pause length.

#### Egg quality

We reported heterosis for traits reflecting egg shape, eggshell, albumen, and yolk qualities. Eggs from both crossbreds showed substantial heterosis for eggshell colour, suggesting that the crossbreds have a lighter brown eggshell colour than the parental mean. This is favourable for some markets where eggs with a light brown colour are more popular than those with a dark brown colour, which is the case in China [2]. For eggshell strength, the YW line showed positive and considerable heterosis at the first four time points, indicating that eggshell of the crossbreds is stronger than the parental mean. The crossbreds showed unfavourable heterosis for Haugh unit in our study, which is consistent with the findings of an earlier study [17]. Haugh unit is positively related with height of the thick albumen and negatively related with egg weight [42]. In the current study, the significant but negative heterosis for Haugh unit may be explained by the significant and positive heterosis for egg weight. For WY, another possible explanation for the negative heterosis for Haugh unit is the significant negative heterosis for albumen height (Additional file 9: Table S15).

### Differences between reciprocal crosses

After adjusting for multiple testing, the reciprocal cross differences were significant only for eggshell strength at 54 weeks of age. However, we found that the degree of heterosis was quite different between reciprocal crosses. Egg weight heterosis ranged from 1.0 to 5.7% for WY and from 4.0 to 9.0% for YW; heterosis for eggshell strength at the early laying stage ranged from 0.9 to 4.8% for WY and from 10.3 to 12.6% for YW. These findings are similar to previously reported results [18, 19]. Sex-linked effects are thought to be one of the factors that affect reciprocal cross differences [43]. Sutherland et al. reported differences between reciprocal crosses for abdominal fat in chickens, and suggested that Z-linked genes may underlie this difference [44]. Other evidence suggested that the effect of genes on the W chromosome depends on the line from which the W chromosome is inherited, which thereby could make a contribution to reciprocal cross differences [45]. In our dataset, WY received the Z chromosome from their WW sire and the W chromosome from their YY dam, while YW received the Z chromosome from their YY sire and the W chromosome from their WW dam. Thus, any effects of sex-linked genes that depend on the breed they originated from are completely confounded with breed effects, and whether Z-linked or W-linked genes are responsible for the reciprocal cross differences cannot be determined. Other plausible explanations for the reciprocal effects in some traits include parent-of-origin specific quantitative trait loci [46] and different breed origin of the mitochondrial DNA [47]. Taken together, all these observations suggest that the decision on which breed to use as the sire line has an impact on the degree of heterosis.

### Heterosis and heritability

Heterosis is expected to be the result from non-additive effects [1]. Narrow sense heritability is defined as the proportion of variability that can be attributed to inherited genetic factors [48]. It has been suggested that a relationship exists between heterosis and heritability, in that lowly-heritable traits benefit the most from outbreeding, which results in the largest amount of heterosis [9, 10]. However, there is relatively limited empirical evidence to support this premise. To investigate this, we calculated the Spearman correlation coefficient between estimates of heterosis and heritability for the traits included in our study. The results suggested that the degree of heterosis expressed across the traits was indeed negatively correlated with the heritability (see Additional file 10: Fig. S5), which is in agreement with the previous statements. This observed negative correlation was, however, only significantly different from zero for the egg production traits. It should be noted that the observed relationship between heterosis and heritabilities may be biased due to the high correlations between traits included (i.e., egg weights at different time points). Nevertheless, our traits covered a broad range of heritabilities (from 0.01 to 0.75), and the estimate of the regression coefficient suggests that with a 0.1 increase in heritability, the expected heterosis could decrease by 0.6% across all investigated traits, and by 3.0% for cumulative egg number and related components traits.

## Conclusions

Heterosis for egg weight and cumulative egg number is substantial and increased with age in crossbreds of Beijing-You and White Leghorn chickens. Crossbreds have better egg production persistency than the superior White Leghorn parent. The degree of heterosis differed significantly between the reciprocal crosses. These findings can contribute to establishing effective poultry breeding schemes that use indigenous and elite lines for niche markets.

### Supplementary Information

**Additional file 1: Table S1:** Percentage of outliers for egg weight traits. The data were filtered by the mean ± three standard deviations to remove the outliers. **Table S2.** Percentage of outliers for egg production traits. The data were filtered by the mean ± three standard deviations to remove the outliers. **Table S3.** Percentage of outliers for egg quality traits. The data were filtered by the mean ± three standard deviations to remove the outliers.

**Additional file 2: Figure S1.** Genetic and phenotypic correlations between the same egg quality trait at different ages.

**Additional file 3: Tables S4.** Heterosis and reciprocal cross differences of egg weight traits for the Dickerson model and comparison between the univariate model and the Dickerson model. **Tables S5.** Heterosis and reciprocal cross differences of egg production traits for the Dickerson model and comparison between the univariate model and the Dickerson model. **Tables S6.** Heterosis and reciprocal cross differences of egg quality traits for the Dickerson model and comparison between the univariate model and the Dickerson model.

**Additional file 4: Table S7.** Heterosis of egg weight traits for multivariate model and comparison between the univariate model and the multivariate model. **Table S8.** Heterosis of egg production traits for multivariate model and comparison between the univariate model and the multivariate model. **Table S9.** Heterosis of egg quality traits for multivariate model and comparison between the univariate model and the multivariate model.

**Additional file 5: Figure S2.** Spearman correlation coefficient between the univariate and the multivariate model for heritabilities (a) and repeatabilities (b).

**Additional file 6: Table S10.** Variances, heritabilities, and repeatabilities of egg weight traits for Beijing-You, White Leghorns, and crossbreds. **Table S11.** Title: Variances, heritabilities, and repeatabilities of egg-production traits for Beijing-You, White Leghorns, and crossbreds. **Table S12.** Variances, heritabilities, and repeatabilities of egg quality traits for Beijing-You, White Leghorns, and crossbreds.

**Additional file 7: Table S13.** Variances for egg weight traits at different ages with the univariate model. **Table S14.** Variances for egg quality traits at different ages with the univariate model.

**Additional file 8: Figure S3.** Heterosis of egg number at separate periods for reciprocal crosses. **Figure S4.** Heterosis of cumulative egg number for reciprocal crosses.

**Additional file 9: Table S15.** Predicted albumen height for the four genetic groups and heterosis for reciprocal crosses.

**Additional file 10:** **Figure S5.** Spearman correlation coefficients between average heterosis and heritability for each trait.

## Data Availability

The datasets used during the current study are available from the corresponding author upon reasonable request.
