# Supplementary material for: Genetic parameters, reciprocal cross differences, and age-related heterosis of egg-laying performance in chickens
Source: Genet Sel Evol. 2023 Dec 7;55:87. doi: 10.1186/s12711-023-00862-7 (PMC10702067; doi:10.1186/s12711-023-00862-7)
Supplement: Supplementary file 1 — Additional file 1: Table S1: Percentage of outliers for egg weight traits. The data were filtered by the mean ± three standard deviations to remove the outliers. Table S2. Percentage of outliers for egg production traits. The data were filtered by the mean ± three standard deviations to remove the outliers. Table S3. Percentage of outliers for egg quality traits. The data were filtered by the mean ± three standard deviations to remove the outliers. [file 12711_2023_862_MOESM1_ESM.docx]

**Additional file 1 Tables S1-S3**

For each trait category, the data were filtered by the mean ± three standard deviations to remove the outliers. The percentage of outliers for each trait are shown in Table S1-S3.

**Table S1. The** **percentage of outliers for egg weight traits**

| Group | FEWt | EWt28 | EWt32 | EWt36 | EWt40 | EWt44 | EWt48 | EWt52 | EWt56 | EWt60 | EWt64 | EWt68 | EWt72 | EWt76 | EWt86 | EWt100 |
| --- | --- | --- | --- | --- | --- | --- | --- | --- | --- | --- | --- | --- | --- | --- | --- | --- |
| WW | 0.55 | 1.49 | 0.57 | 0.64 | 0.17 | 0.28 | 0.37 | 0.39 | 0.40 | 0.89 | 0.45 | 0.44 | 1.22 | 0.29 | 0.87 | 0.74 |
| YY | 1.45 | 1.47 | 0.97 | 0.48 | 0.32 | 0.89 | 0.96 | 0.16 | 0.75 | 0.00 | 0.95 | 0.46 | 0.44 | 1.59 | 0.50 | 1.28 |
| WY | 1.78 | 2.01 | 1.22 | 0.84 | 0.32 | 0.35 | 0.16 | 0.16 | 0.00 | 0.00 | 0.20 | 0.28 | 0.77 | 0.97 | 1.82 | 0.00 |
| YW | 1.42 | 1.76 | 0.44 | 0.68 | 0.24 | 0.36 | 0.52 | 0.13 | 0.14 | 0.17 | 0.17 | 0.40 | 0.00 | 0.43 | 0.18 | 0.58 |
| Total | 1.33 | 1.69 | 0.80 | 0.66 | 0.26 | 0.47 | 0.50 | 0.20 | 0.30 | 0.26 | 0.41 | 0.39 | 0.54 | 0.81 | 0.83 | 0.59 |

FEWt: weight for the first three egg, EWtX: egg weight at X weeks of age.

**Table S2. The percentage of outliers for egg production traits**

| Group | AFE | OP | EN43 | NC43 | ACL43 | APL43 | EN72 | NC72 | ACL72 | APL72 | EN100 | NC100 | ACL100 | APL100 |
| --- | --- | --- | --- | --- | --- | --- | --- | --- | --- | --- | --- | --- | --- | --- |
| WW | 1.59 | 1.80 | 3.30 | 0.47 | 1.89 | 3.32 | 3.01 | 0.60 | 1.81 | 1.20 | 1.88 | 0.00 | 1.25 | 1.25 |
| YY | 0.68 | 0.77 | 3.33 | 0.00 | 1.48 | 1.49 | 3.03 | 2.53 | 1.01 | 1.01 | 2.65 | 1.59 | 0.53 | 1.59 |
| WY | 1.07 | 0.00 | 2.59 | 0.86 | 1.29 | 1.73 | 4.17 | 0.52 | 1.04 | 1.04 | 3.28 | 1.64 | 1.09 | 0.55 |
| YW | 0.00 | 0.00 | 2.40 | 0.00 | 2.00 | 2.40 | 2.94 | 0.49 | 2.45 | 1.47 | 2.65 | 0.53 | 2.12 | 1.59 |
| Total | 0.80 | 1.52 | 2.90 | 0.31 | 1.66 | 2.19 | 3.29 | 1.05 | 1.58 | 1.18 | 2.64 | 0.97 | 1.25 | 1.25 |

AFE: age at first egg, OP: oviposition period, ENX: cumulative egg number till X weeks of age, NCX: number of clutches till X weeks of age, ACLX: average clutch length till X weeks of age, APLX: average pause length till X weeks of age.

**Table S3. The percentage of outliers for egg quality traits**

| Weeks of age | Group | ESI | ESC(%) | ESS(kg/cm2) | EST(mm) | ESR | YR | YC | HU |
| --- | --- | --- | --- | --- | --- | --- | --- | --- | --- |
| 32 | WW | 1.05 | 0.30 | 0.76 | 0.61 | 1.33 | 0.77 | 0.19 | 1.33 |
|  | YY | 1.54 | 0.83 | 1.08 | 0.24 | 0.61 | 0.89 | 0.12 | 1.21 |
|  | WY | 1.34 | 0.94 | 1.75 | 1.34 | 1.90 | 1.80 | 0.54 | 0.95 |
|  | YW | 0.54 | 0.32 | 0.98 | 0.87 | 1.55 | 0.68 | 0.11 | 1.88 |
|  | Total | 1.10 | 0.60 | 1.14 | 0.76 | 1.33 | 1.03 | 0.23 | 1.37 |
| 54 | WW | 1.32 | 0.95 | 0.95 | 1.32 | 1.34 | 1.15 | 2.48 | 1.33 |
|  | YY | 1.63 | 0.18 | 0.72 | 0.72 | 1.28 | 0.94 | 1.10 | 1.47 |
|  | WY | 1.60 | 0.81 | 1.29 | 1.60 | 1.46 | 0.99 | 0.48 | 1.78 |
|  | YW | 2.91 | 0.28 | 1.38 | 1.37 | 1.39 | 0.70 | 0.97 | 1.25 |
|  | Total | 1.94 | 0.54 | 1.11 | 1.27 | 1.37 | 0.93 | 1.20 | 1.45 |
| 72 | WW | 1.20 | 0.96 | 0.48 | 0.48 | 0.99 | 0.99 | 0.24 | 1.96 |
|  | YY | 1.08 | 0.22 | 1.09 | 0.43 | 1.34 | 0.23 | 0.00 | 2.44 |
|  | WY | 1.13 | 0.00 | 1.32 | 1.32 | 1.92 | 0.59 | 0.00 | 1.34 |
|  | YW | 1.06 | 0.61 | 0.91 | 1.06 | 1.54 | 1.42 | 0.30 | 2.61 |
|  | Total | 1.11 | 0.43 | 0.97 | 0.87 | 1.49 | 0.85 | 0.14 | 2.12 |
| 86 | WW | 0.58 | 1.44 | 0.00 | 0.61 | 1.56 | 1.20 | 0.00 | 2.06 |
|  | YY | 0.49 | 0.00 | 0.25 | 1.22 | 1.00 | 1.27 | 0.00 | 2.53 |
|  | WY | 0.39 | 0.59 | 0.60 | 1.91 | 1.52 | 1.67 | 0.22 | 0.61 |
|  | YW | 0.52 | 0.70 | 0.52 | 1.33 | 0.93 | 1.25 | 0.00 | 1.77 |
|  | Total | 0.49 | 0.65 | 0.38 | 1.31 | 1.23 | 1.36 | 0.06 | 1.68 |
| 100 | WW | 1.72 | 0.49 | 0.00 | 0.00 | 0.74 | 1.54 | 0.00 | 1.28 |
|  | YY | 1.27 | 0.64 | 0.00 | 0.35 | 1.06 | 1.15 | 0.34 | 1.70 |
|  | WY | 1.29 | 1.29 | 0.00 | 0.65 | 1.84 | 2.05 | 0.23 | 0.68 |
|  | YW | 1.17 | 0.97 | 0.40 | 0.39 | 0.78 | 0.42 | 0.00 | 2.30 |
|  | Total | 1.36 | 0.88 | 0.12 | 0.36 | 1.10 | 1.27 | 0.13 | 1.50 |

ESI: egg shape index, ESC: eggshell colour, ESS: eggshell strength, EST: eggshell thickness, ESR: eggshell ratio, YR: yolk ratio, YC: yolk colour, HU: Haugh unit.
