# Supplementary material for: Genetic parameters, reciprocal cross differences, and age-related heterosis of egg-laying performance in chickens
Source: Genet Sel Evol. 2023 Dec 7;55:87. doi: 10.1186/s12711-023-00862-7 (PMC10702067; doi:10.1186/s12711-023-00862-7)
Supplement: Supplementary file 2 — Additional file 2: Figure S1. Genetic and phenotypic correlations between the same egg quality trait at different ages. [file 12711_2023_862_MOESM2_ESM.docx]

**Additional file 2 Figure S1**

Genetic and phenotypic correlations between the same egg quality trait at different ages are shown in Figure S1.


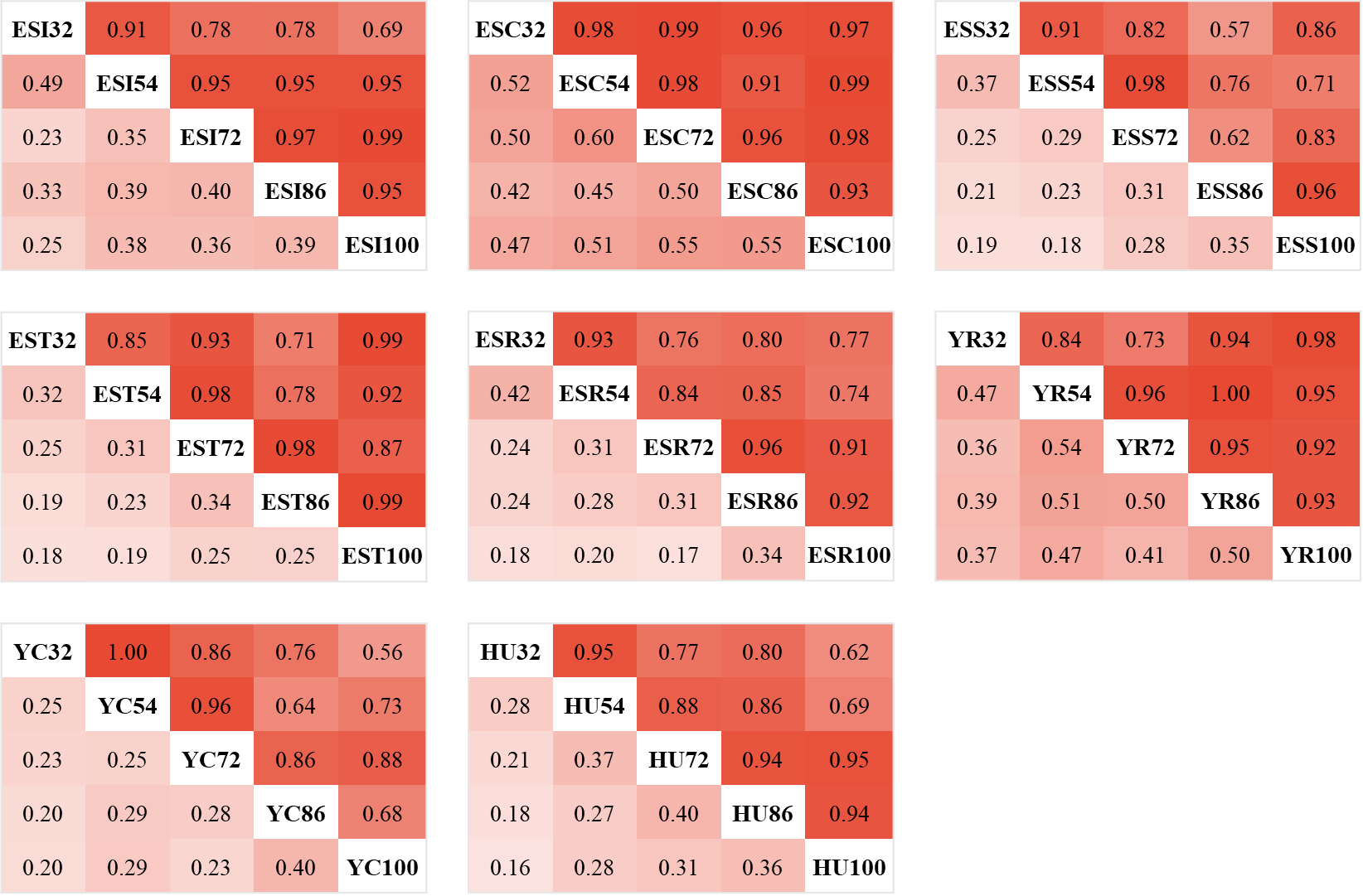


**Figure S1. Genetic and phenotypic correlations between the same egg quality trait at different ages.**

ESIX: egg shape index at X weeks of age, ESCX: eggshell colour at X weeks of age, ESSX: eggshell strength at X weeks of age, ESTX: eggshell thickness at X weeks of age, ESRX: eggshell ratio at X weeks of age, YRX; yolk ratio at X weeks of age, YCX: yolk colour at X weeks of age, HUX: Haugh unit at X weeks of age.

The upper triangle are genetic correlations and lower triangle are phenotypic correlations.
