# Supplementary material for: Genetic parameters, reciprocal cross differences, and age-related heterosis of egg-laying performance in chickens
Source: Genet Sel Evol. 2023 Dec 7;55:87. doi: 10.1186/s12711-023-00862-7 (PMC10702067; doi:10.1186/s12711-023-00862-7)
Supplement: Supplementary file 3 — Additional file 3: Tables S4. Heterosis and reciprocal cross differences of egg weight traits for the Dickerson model and comparison between the univariate model and the Dickerson model. Tables S5. Heterosis and reciprocal cross differences of egg production traits for the Dickerson model and comparison between the univariate model and the Dickerson model. Tables S6. Heterosis and reciprocal cross differences of egg quality traits for the Dickerson model and comparison between the univariate model and the Dickerson model. [file 12711_2023_862_MOESM3_ESM.docx]

**Additional file 3 Tables S4-S6**

Heterosis and reciprocal cross differences of egg-laying performance traits for the Dickerson model and comparison between univariate model and the Dickerson model are shown in Table S4-S6.

**Table S4. Heterosis and reciprocal cross differences of egg weight traits for the Dickerson model and comparison between the univariate model and the Dickerson model**

| Traits | Dickerson model | | Difference between the univariate and the Dickerson model | |
| --- | --- | --- | --- | --- |
|  | Heterosis | Reciprocal cross differences | Heterosis | Reciprocal cross differences |
| FEWt | 1.34 | 0.89 | -1.88E-04 | 0.00E+00 |
| EWt28 | 1.68 | 0.69 | -5.00E-04 | 0.00E+00 |
| EWt32 | 1.71 | 0.39 | -4.00E-04 | 0.00E+00 |
| EWt36 | 1.90 | 0.66 | 0.00E+00 | 1.00E-04 |
| EWt40 | 2.06 | 0.57 | -5.00E-04 | 2.00E-04 |
| EWt44 | 2.05 | 0.70 | 5.00E-04 | -2.00E-04 |
| EWt48 | 2.29 | 0.70 | -2.00E-04 | 2.00E-04 |
| EWt52 | 2.49 | 0.87 | -3.50E-04 | 1.00E-04 |
| EWt56 | 2.65 | 1.01 | 0.00E+00 | 5.00E-04 |
| EWt60 | 2.72 | 0.93 | -5.00E-05 | -2.00E-04 |
| EWt64 | 2.84 | 0.85 | 2.00E-04 | 2.00E-04 |
| EWt68 | 3.10 | 0.83 | 0.00E+00 | -1.00E-04 |
| EWt72 | 3.21 | 0.80 | 0.00E+00 | -1.00E-04 |
| EWt76 | 3.40 | 0.47 | 4.50E-04 | -5.00E-05 |
| EWt86 | 3.80 | 0.54 | 0.00E+00 | 1.00E-04 |
| EWt100 | 4.17 | 1.27 | 5.00E-04 | 0.00E+00 |

FEWt: weight for the first three egg, EWtX: egg weight at X weeks of age.

**Table S5. Heterosis and reciprocal cross differences of egg production traits for proposed model and comparison between the univariate model and the Dickerson model**

| Traits | Dickerson model | | Difference between the univariate and the Dickerson model | |
| --- | --- | --- | --- | --- |
|  | Heterosis | Reciprocal cross differences | Heterosis | Reciprocal cross differences |
| AFE | -2.72 | 0.75 | 5.00E-04 | -1.00E-04 |
| OP | -0.08 | 0.08 | -4.00E-05 | 1.00E-05 |
| EN43 | 2.11 | -0.74 | 1.50E-03 | 2.00E-04 |
| NC43 | 0.73 | 0.29 | 2.00E-04 | 0.00E+00 |
| ACL43 | -2.38 | -0.19 | -1.00E-04 | 0.00E+00 |
| APL43 | -0.04 | 0.00 | -5.00E-06 | 0.00E+00 |
| EN72 | 10.36 | -2.33 | 1.00E-03 | 0.00E+00 |
| NC72 | 0.62 | -0.54 | 2.20E-03 | -2.00E-04 |
| ACL72 | -1.35 | -0.05 | 4.60E-04 | 0.00E+00 |
| APL72 | -0.21 | 0.04 | -1.00E-05 | 0.00E+00 |
| EN100 | 32.49 | -4.90 | 8.00E-03 | 0.00E+00 |
| NC100 | 8.03 | -2.20 | -1.00E-03 | 0.00E+00 |
| ACL100 | -0.68 | -0.03 | -1.90E-04 | 0.00E+00 |
| APL100 | -0.64 | -0.01 | -5.00E-06 | -1.00E-06 |

AFE: age at first egg, OP: oviposition period, ENX: cumulative egg number till X weeks of age, NCX: number of clutches till X weeks of age, ACLX: average clutch length till X weeks of age, APLX: average pause length till X weeks of age.

**Table S6. Heterosis and reciprocal cross differences of egg quality traits for the Dickerson model and comparison between the univariate model and the Dickerson model**

| Traits | Dickerson model | | Difference between the univariate and the Dickerson model | |
| --- | --- | --- | --- | --- |
|  | Heterosis | Reciprocal cross differences | Heterosis | Reciprocal cross differences |
| ESI32 | -0.43 | 0.09 | -1.00E-04 | 0.00E+00 |
| ESC32 | 4.35 | -0.79 | -5.00E-04 | -1.00E-04 |
| ESS32 | 0.28 | 0.10 | -4.00E-05 | -1.00E-05 |
| EST32 | 0.01 | 0.00 | -1.00E-06 | 0.00E+00 |
| ESR32 | 0.19 | 0.08 | -3.50E-05 | 2.00E-05 |
| YR32 | 0.56 | -0.01 | -1.30E-04 | 0.00E+00 |
| YC32 | 0.06 | -0.20 | -3.00E-05 | 0.00E+00 |
| HU32 | -2.11 | -0.26 | 1.50E-04 | -5.00E-05 |
| ESI54 | -0.34 | 0.04 | 2.50E-05 | -5.00E-06 |
| ESC54 | 3.87 | -1.11 | 5.00E-04 | -5.00E-04 |
| ESS54 | 0.22 | 0.19 | -3.50E-05 | 0.00E+00 |
| EST54 | 0.01 | 0.01 | 5.00E-06 | -2.00E-06 |
| ESR54 | 0.16 | 0.12 | -3.00E-05 | 0.00E+00 |
| YR54 | 0.49 | 0.03 | -2.20E-04 | 0.00E+00 |
| YC54 | 0.07 | -0.03 | 3.50E-05 | -5.00E-06 |
| HU54 | -2.61 | 0.45 | -5.00E-05 | -5.00E-05 |
| ESI72 | 0.05 | 0.02 | -1.50E-05 | -5.00E-06 |
| ESC72 | 3.67 | -0.41 | -1.85E-03 | 5.00E-05 |
| ESS72 | 0.24 | 0.12 | -4.25E-05 | 0.00E+00 |
| EST72 | 0.02 | 0.00 | 6.00E-06 | 0.00E+00 |
| ESR72 | 0.27 | 0.04 | -2.00E-05 | 0.00E+00 |
| YR72 | 0.12 | 0.10 | -3.50E-04 | -5.00E-05 |
| YC72 | -0.11 | -0.02 | 5.50E-05 | 5.00E-06 |
| HU72 | -2.86 | 1.44 | 1.00E-04 | -5.00E-04 |
| ESI86 | 0.44 | 0.16 | -5.00E-05 | -5.00E-05 |
| ESC86 | 4.81 | -0.55 | 0.00E+00 | -1.00E-04 |
| ESS86 | 0.12 | 0.06 | 0.00E+00 | 0.00E+00 |
| EST86 | 0.01 | 0.00 | -2.00E-06 | 0.00E+00 |
| ESR86 | 0.25 | 0.06 | 5.00E-05 | -1.00E-05 |
| YR86 | 0.14 | 0.23 | -1.50E-04 | -5.00E-05 |
| YC86 | -0.20 | 0.11 | 0.00E+00 | -5.00E-05 |
| HU86 | -4.73 | 1.35 | 0.00E+00 | -5.00E-04 |
| ESI100 | -0.29 | -0.10 | 1.00E-04 | 0.00E+00 |
| ESC100 | 4.47 | -0.65 | -1.00E-03 | 2.00E-04 |
| ESS100 | 0.10 | 0.00 | -5.75E-05 | 5.00E-07 |
| EST100 | 0.01 | 0.01 | -2.00E-06 | 0.00E+00 |
| ESR100 | 0.16 | -0.03 | 2.50E-05 | 5.00E-06 |
| YR100 | 0.53 | 0.01 | -2.10E-04 | 0.00E+00 |
| YC100 | 0.21 | 0.12 | 0.00E+00 | -5.00E-05 |
| HU100 | -5.22 | 1.71 | -5.00E-04 | -5.00E-04 |

ESIX: egg shape index at X weeks of age, ESCX: eggshell colour at X weeks of age, ESSX: eggshell strength at X weeks of age, ESTX: eggshell thickness at X weeks of age, ESRX: eggshell ratio at X weeks of age, YRX: yolk ratio at X weeks of age, YCX: yolk colour at X weeks of age, HUX: Haugh unit at X weeks of age.
