# Supplementary material for: Genetic parameters, reciprocal cross differences, and age-related heterosis of egg-laying performance in chickens
Source: Genet Sel Evol. 2023 Dec 7;55:87. doi: 10.1186/s12711-023-00862-7 (PMC10702067; doi:10.1186/s12711-023-00862-7)
Supplement: Supplementary file 4 — Additional file 4: Table S7. Heterosis of egg weight traits for multivariate model and comparison between the univariate model and the multivariate model. Table S8. Heterosis of egg production traits for multivariate model and comparison between the univariate model and the multivariate model. Table S9. Heterosis of egg quality traits for multivariate model and comparison between the univariate model and the multivariate model. [file 12711_2023_862_MOESM4_ESM.docx]

**Additional file 4 Tables S7-S9**

Heterosis of egg-laying performance traits for multivariate model and comparison between the univariate model and the multivariate model are shown in Table S7-S9.

**Table S7. Heterosis of egg weight traits for multivariate model and comparison between the univariate model and the multivariate model**

| Traits | Multivariate model | | | Difference between univariate and multivariate model | | | | |
| --- | --- | --- | --- | --- | --- | --- | --- | --- |
|  | H%(WY) | H%(YW) | Reciprocal cross differences (%) | Heritability (*h^2^*) | Repeatability (*r*) | H%(WY) | H%(YW) | Reciprocal cross differences |
| FEWt | 1.00 | 4.89 | -3.89 | -0.01 | 0.00 | -0.02 | -0.23 | 0.20 |
| EWt28 | 1.81 | 4.04 | -2.23 | 0.00 | 0.00 | -0.07 | -0.46 | 0.39 |
| EWt32 | 2.20 | 3.42 | -1.22 | -0.02 | -0.01 | -0.29 | -0.54 | 0.25 |
| EWt36 | 1.90 | 3.91 | -2.01 | 0.01 | 0.00 | -0.28 | -0.48 | 0.20 |
| EWt40 | 2.27 | 3.99 | -1.72 | 0.00 | 0.00 | -0.39 | -0.53 | 0.14 |
| EWt44 | 2.15 | 4.28 | -2.13 | 0.01 | 0.00 | -0.26 | -0.44 | 0.18 |
| EWt48 | 2.23 | 4.60 | -2.36 | -0.03 | 0.00 | -0.48 | -0.58 | 0.10 |
| EWt52 | 2.15 | 4.99 | -2.84 | -0.01 | 0.00 | -0.61 | -0.71 | 0.10 |
| EWt56 | 2.28 | 5.58 | -3.30 | -0.03 | 0.00 | -0.51 | -0.60 | 0.09 |
| EWt60 | 2.51 | 5.25 | -2.74 | 0.01 | 0.00 | -0.45 | -0.50 | 0.04 |
| EWt64 | 2.81 | 5.73 | -2.92 | 0.03 | 0.00 | -0.46 | -0.29 | -0.17 |
| EWt68 | 3.28 | 5.92 | -2.64 | 0.01 | 0.00 | -0.51 | -0.51 | 0.00 |
| EWt72 | 3.72 | 6.63 | -2.91 | -0.04 | -0.01 | -0.32 | -0.10 | -0.23 |
| EWt76 | 4.69 | 6.49 | -1.80 | -0.02 | -0.01 | -0.16 | -0.17 | 0.02 |
| EWt86 | 5.25 | 6.93 | -1.69 | -0.07 | -0.01 | -0.44 | -0.32 | -0.12 |
| EWt100 | 4.86 | 9.05 | -4.19 | 0.04 | 0.00 | -0.08 | 0.08 | -0.16 |

FEWt: weight for the first three egg, EWtX: egg weight at X weeks of age.

H% (WY): Percent heterosis for WY, the percentage of performance of WY being better than the average performance of the two parental lines, H% (YW): Percent heterosis for YW, the percentage of performance of YW being better than the average performance of the two parental lines.

**Table S8. Heterosis of egg production traits for multivariate model and comparison between the univariate model and the multivariate model**

| Traits | Multivariate model | | | Difference between univariate and multivariate model | | | |
| --- | --- | --- | --- | --- | --- | --- | --- |
|  | H%(WY) | H%(YW) | Reciprocal cross differences (%) | Heritability (*h^2^*) | H%(WY) | H%(YW) | Reciprocal cross differences |
| AFE | -1.83 | -1.11 | -0.72 | 0.07 | 0.17 | 0.18 | -0.01 |
| OP | -0.46 | 0.25 | -0.71 | 0.09 | 0.15 | 0.12 | 0.03 |
| EN43 | 2.04 | 1.12 | 0.93 | 0.01 | -0.44 | -0.32 | -0.11 |
| NC43 | 2.83 | 7.27 | -4.44 | -0.03 | 0.14 | 1.72 | -1.58 |
| ACL43 | -25.47 | -29.04 | 3.57 | 0.23 | -0.46 | -0.25 | -0.21 |
| APL43 | -3.16 | -4.96 | 1.80 | 0.03 | -0.01 | -0.07 | 0.06 |
| EN72 | 4.73 | 2.33 | 2.40 | -0.05 | -0.41 | -0.35 | -0.07 |
| NC72 | 3.25 | 0.14 | 3.11 | 0.01 | 1.24 | 1.50 | -0.25 |
| ACL72 | -22.00 | -22.49 | 0.48 | 0.19 | -0.91 | -0.26 | -0.65 |
| APL72 | -15.03 | -11.97 | -3.06 | 0.00 | 0.28 | 0.59 | -0.31 |
| EN100 | 11.37 | 8.75 | 2.62 | -0.03 | -0.18 | 0.25 | -0.43 |
| NC100 | 12.62 | 7.55 | 5.07 | 0.08 | 1.08 | 1.43 | -0.34 |
| ACL100 | -15.26 | -15.45 | 0.18 | 0.18 | -1.04 | -0.37 | -0.67 |
| APL100 | -23.39 | -22.62 | -0.76 | 0.06 | 1.20 | -0.33 | 1.52 |

AFE: age at first egg, OP: oviposition period, ENX: cumulative egg number till X weeks of age, NCX: number of clutches till X weeks of age, ACLX: average clutch length till X weeks of age, APLX: average pause length till X weeks of age.

H% (WY): Percent heterosis for WY, the percentage of performance of WY being better than the average performance of the two parental lines, H% (YW): Percent heterosis for YW, the percentage of performance of YW being better than the average performance of the two parental lines.

**Table S9. Heterosis of egg quality traits for multivariate model and comparison between the univariate model and the multivariate model**

| Traits | Multivariate model | | | Difference between univariate and multivariate model | | | | |
| --- | --- | --- | --- | --- | --- | --- | --- | --- |
|  | H%(WY) | H%(YW) | Reciprocal cross differences (%) | Heritability (*h^2^*) | Repeatability (*r*) | H%(WY) | H%(YW) | Reciprocal cross differences |
| ESI32 | -0.63 | -0.35 | -0.28 | -0.04 | 0.01 | 0.04 | 0.09 | -0.05 |
| ESC32 | 7.55 | 5.11 | 2.44 | 0.07 | 0.01 | -0.62 | -0.48 | -0.14 |
| ESS32 | 5.41 | 10.91 | -5.50 | 0.00 | 0.00 | 0.58 | 0.60 | -0.02 |
| EST32 | 1.20 | 2.95 | -1.76 | 0.00 | 0.00 | 0.36 | 0.22 | 0.14 |
| ESR32 | 1.58 | 2.95 | -1.37 | -0.02 | 0.00 | 0.38 | 0.41 | -0.03 |
| YR32 | 1.98 | 2.26 | -0.28 | 0.08 | 0.02 | 0.04 | 0.11 | -0.07 |
| YC32 | 4.78 | -1.40 | 6.18 | 0.01 | -0.03 | 0.20 | 0.15 | 0.05 |
| HU32 | -2.77 | -3.40 | 0.64 | -0.02 | 0.00 | -0.07 | -0.06 | -0.01 |
| ESI54 | -0.49 | -0.48 | -0.01 | 0.05 | 0.03 | 0.05 | 0.14 | -0.09 |
| ESC54 | 7.55 | 3.64 | 3.92 | 0.10 | 0.02 | -0.30 | -0.28 | -0.02 |
| ESS54 | 1.32 | 12.70 | -11.38 | -0.01 | -0.01 | 0.44 | 0.13 | 0.31 |
| EST54 | 0.86 | 4.91 | -4.05 | 0.02 | 0.01 | 0.27 | 0.22 | 0.05 |
| ESR54 | 0.77 | 3.14 | -2.37 | 0.00 | -0.02 | 0.28 | 0.22 | 0.06 |
| YR54 | 1.32 | 1.97 | -0.65 | -0.01 | 0.00 | -0.09 | 0.04 | -0.13 |
| YC54 | 1.70 | -0.78 | 2.49 | -0.04 | 0.01 | -0.20 | -0.36 | 0.15 |
| HU54 | -4.06 | -2.74 | -1.32 | 0.05 | -0.01 | -0.09 | 0.08 | -0.17 |
| ESI72 | 0.00 | -0.07 | 0.08 | -0.04 | 0.01 | 0.02 | 0.02 | 0.00 |
| ESC72 | 6.36 | 5.19 | 1.17 | 0.02 | 0.01 | -0.45 | -0.42 | -0.03 |
| ESS72 | 3.82 | 11.29 | -7.47 | 0.03 | 0.00 | 0.24 | -0.07 | 0.31 |
| EST72 | 4.28 | 6.66 | -2.38 | 0.10 | 0.02 | 0.14 | 0.08 | 0.06 |
| ESR72 | 2.72 | 3.96 | -1.24 | 0.02 | 0.00 | 0.13 | 0.05 | 0.08 |
| YR72 | 0.16 | 0.88 | -0.72 | 0.07 | 0.02 | 0.21 | 0.14 | 0.07 |
| YC72 | -1.05 | -0.67 | -0.38 | 0.00 | 0.02 | 0.18 | -0.09 | 0.27 |
| HU72 | -5.70 | -2.61 | -3.09 | 0.02 | -0.01 | -0.07 | 0.19 | -0.26 |
| ESI86 | 0.42 | 0.55 | -0.13 | -0.04 | 0.00 | 0.02 | -0.09 | 0.11 |
| ESC86 | 9.46 | 6.89 | 2.57 | 0.00 | 0.02 | -0.01 | -0.07 | 0.05 |
| ESS86 | 2.91 | 6.46 | -3.56 | -0.04 | 0.00 | 0.74 | 0.21 | 0.53 |
| EST86 | 3.46 | 5.96 | -2.50 | -0.02 | 0.01 | 0.20 | 0.05 | 0.15 |
| ESR86 | 2.47 | 3.44 | -0.96 | -0.01 | 0.00 | 0.26 | -0.15 | 0.40 |
| YR86 | -0.29 | 1.23 | -1.52 | -0.01 | 0.00 | 0.00 | 0.08 | -0.08 |
| YC86 | -4.86 | -2.03 | -2.82 | 0.03 | 0.00 | 0.24 | -0.25 | 0.49 |
| HU86 | -9.09 | -4.84 | -4.25 | -0.01 | -0.01 | -0.38 | 0.16 | -0.54 |
| ESI100 | -0.21 | -0.65 | 0.44 | -0.03 | 0.01 | 0.15 | 0.17 | -0.02 |
| ESC100 | 9.35 | 6.53 | 2.82 | 0.06 | 0.03 | -0.25 | -0.14 | -0.10 |
| ESS100 | 3.32 | 1.28 | 2.04 | 0.01 | 0.00 | 0.29 | 0.20 | 0.09 |
| EST100 | 0.44 | 4.23 | -3.79 | 0.04 | 0.00 | 0.25 | 0.04 | 0.21 |
| ESR100 | 2.20 | 0.91 | 1.29 | 0.03 | 0.01 | 0.26 | -0.05 | 0.31 |
| YR100 | 1.69 | 2.14 | -0.45 | 0.05 | 0.00 | 0.09 | -0.08 | 0.17 |
| YC100 | 0.25 | 5.31 | -5.06 | 0.01 | -0.01 | -0.49 | -0.81 | 0.32 |
| HU100 | -9.94 | -4.49 | -5.44 | 0.00 | -0.01 | -0.12 | 0.54 | -0.66 |

ESIX: egg shape index at X weeks of age, ESCX: eggshell colour at X weeks of age, ESSX: eggshell strength at X weeks of age, ESTX: eggshell thickness at X weeks of age, ESRX: eggshell ratio at X weeks of age, YRX: yolk ratio at X weeks of age, YCX: yolk colour at X weeks of age, HUX: Haugh unit at X weeks of age.

H% (WY): Percent heterosis for WY, the percentage of performance of WY being better than the average performance of the two parental lines, H% (YW): Percent heterosis for YW, the percentage of performance of YW being better than the average performance of the two parental lines.
