# Supplementary material for: Genetic parameters, reciprocal cross differences, and age-related heterosis of egg-laying performance in chickens
Source: Genet Sel Evol. 2023 Dec 7;55:87. doi: 10.1186/s12711-023-00862-7 (PMC10702067; doi:10.1186/s12711-023-00862-7)
Supplement: Supplementary file 5 — Additional file 5: Figure S2. Spearman correlation coefficient between the univariate and the multivariate model for heritabilities (a) and repeatabilities (b). [file 12711_2023_862_MOESM5_ESM.docx]

**Additional file 5 Figure S2**

The relationship for heritabilities and repeatabilities between the univariate and the multivariate models are shown in Figure S2. The relationship was estimated with the Spearman correlation coefficient using “ggpubr” R-package (<https://www.r-project.org/>). Across all detected traits, the squared correlation coefficient for heritabilities is 0.914, and the squared correlation coefficient for repeatabilities is 0.996.

**a**

**b**


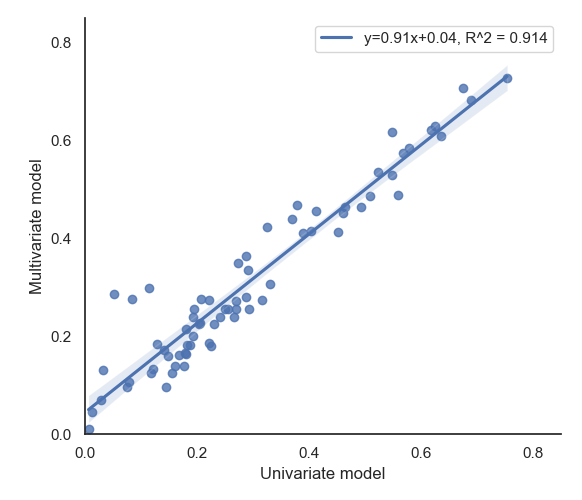

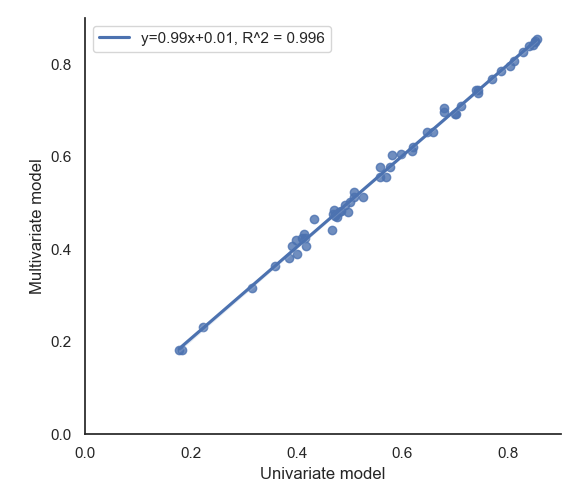


**Figure S2. Spearman correlation coefficient between the univariate and the multivariate model for heritabilities (a) and repeatabilities (b).**
