# Supplementary material for: Genetic parameters, reciprocal cross differences, and age-related heterosis of egg-laying performance in chickens
Source: Genet Sel Evol. 2023 Dec 7;55:87. doi: 10.1186/s12711-023-00862-7 (PMC10702067; doi:10.1186/s12711-023-00862-7)
Supplement: Supplementary file 6 — Additional file 6: Table S10. Variances, heritabilities, and repeatabilities of egg weight traits for Beijing-You, White Leghorns, and crossbreds. Table S11. Title: Variances, heritabilities, and repeatabilities of egg-production traits for Beijing-You, White Leghorns, and crossbreds. Table S12. Variances, heritabilities, and repeatabilities of egg quality traits for Beijing-You, White Leghorns, and crossbreds. [file 12711_2023_862_MOESM6_ESM.docx]

**Additional file 6 Tables S10-S12**

Variances, heritabilities, and repeatabilities of egg-laying performance traits for Beijing-You, White Leghorns, and crossbreds are shown in Table S10-S12, respectively.

**Table S10.** **Variances, heritabilities, and repeatabilities of egg weight traits for Beijing-You, White Leghorns, and crossbreds**

| **Trait** | **Beijing-You** | | | **White Leghorns** | | | **Crossbreds** | | |
| --- | --- | --- | --- | --- | --- | --- | --- | --- | --- |
|  | **Phenotypic variance (**$\boldsymbol{\sigma}_{\boldsymbol{p}}^{\boldsymbol{2}}$**)** | **Heritability (*h^2^*)** | **Repeatability (*****r*)** | **Phenotypic variance (**$\boldsymbol{\sigma}_{\boldsymbol{p}}^{\boldsymbol{2}}$**)** | **Heritability (*h^2^*)** | **Repeatability (*r*)** | **Phenotypic variance (**$\boldsymbol{\sigma}_{\boldsymbol{p}}^{\boldsymbol{2}}$**)** | **Heritability (*h^2^*)** | **Repeatability (*r*)** |
| FEWt | 17.58 | 0.34 | 0.54 | 18.13 | 0.03 | 0.40 | 21.80 | 0.34 | 0.47 |
| EWt28 | 15.46 | 0.44 | 0.64 | 11.32 | 0.27 | 0.78 | 12.52 | 0.55 | 0.72 |
| EWt32 | 12.78 | 0.67 | 0.72 | 13.84 | 0.21 | 0.67 | 13.42 | 0.52 | 0.69 |
| EWt36 | 15.21 | 0.85 | 0.85 | 15.42 | 0.53 | 0.84 | 13.95 | 0.56 | 0.86 |
| EWt40 | 15.80 | 0.83 | 0.83 | 16.06 | 0.36 | 0.84 | 16.01 | 0.64 | 0.86 |
| EWt44 | 15.82 | 0.75 | 0.81 | 13.97 | 0.38 | 0.67 | 17.17 | 0.58 | 0.78 |
| EWt48 | 17.47 | 0.86 | 0.86 | 18.52 | 0.67 | 0.81 | 17.36 | 0.69 | 0.85 |
| EWt52 | 18.91 | 0.78 | 0.83 | 16.99 | 0.52 | 0.77 | 17.77 | 0.70 | 0.81 |
| EWt56 | 19.49 | 0.57 | 0.79 | 18.37 | 0.31 | 0.81 | 19.18 | 0.75 | 0.85 |
| EWt60 | 19.04 | 0.67 | 0.86 | 16.46 | 0.13 | 0.80 | 21.41 | 0.61 | 0.84 |
| EWt64 | 18.22 | 0.69 | 0.87 | 19.87 | 0.63 | 0.83 | 21.60 | 0.74 | 0.85 |
| EWt68 | 22.55 | 0.58 | 0.73 | 19.49 | 0.46 | 0.77 | 22.02 | 0.62 | 0.82 |
| EWt72 | 22.42 | 0.27 | 0.58 | 18.93 | 0.26 | 0.68 | 22.28 | 0.54 | 0.75 |
| EWt76 | 22.28 | 0.45 | 0.83 | 17.28 | 0.23 | 0.66 | 24.87 | 0.65 | 0.83 |
| EWt86 | 29.78 | 0.39 | 0.78 | 25.74 | 0.38 | 0.76 | 27.56 | 0.58 | 0.71 |
| EWt100 | 27.08 | 0.74 | 0.74 | 25.81 | 0.53 | 0.73 | 31.81 | 0.31 | 0.75 |

FEWt: average weight for the first three eggs, EWtX: egg weight at X weeks of age.

For Beijing-You chickens, standard errors ranged from 0.01 to 0.25 for heritabilities, and from 0.01 to 0.05 for repeatabilities. For White Leghorn chickens, standard errors ranged from 0.07 to 0.27 for heritabilities, and from 0.02 to 0.05 for repeatabilities. For crossbreds, standard errors ranged from 0.07 to 0.13 for heritabilities, and from 0.01 to 0.03 for repeatabilities

**Table S11. Variances, and heritabilities of egg production traits for Beijing-You, White Leghorns, and crossbreds**

| **Trait** | **Beijing-You** | | **White Leghorns** | | **Crossbreds** | |
| --- | --- | --- | --- | --- | --- | --- |
|  | **Phenotypic variance (**$\boldsymbol{\sigma}_{\boldsymbol{p}}^{\boldsymbol{2}}$**)** | **Heritability (*h^2^*)** | **Phenotypic variance (**$\boldsymbol{\sigma}_{\boldsymbol{p}}^{\boldsymbol{2}}$**)** | **Heritability (*h^2^*)** | **Phenotypic variance (**$\boldsymbol{\sigma}_{\boldsymbol{p}}^{\boldsymbol{2}}$**)** | **Heritability (*h^2^*)** |
| AFE | 97.42 | 0.56 | 61.87 | 0.31 | 84.55 | 0.74 |
| OP | 1.39 | 0.36 | 0.23 | 0.53 | 0.80 | 0.55 |
| EN43 | 182.75 | 0^*^ | 196.89 | 0.21 | 155.74 | 0.22 |
| NC43 | 59.53 | 0.52 | 37.23 | 0.45 | 41.15 | 0.43 |
| ACL43 | 2.00 | 0.44 | 84.88 | 0.28 | 8.66 | 0.31 |
| APL43 | 0.18 | 0.05 | 0.29 | 0.06 | 0.15 | 0.03 |
| EN72 | 1210.20 | 0.05 | 1777.90 | 0^*^ | 866.57 | 0.21 |
| NC72 | 288.50 | 0.53 | 178.67 | 0.21 | 244.30 | 0.41 |
| ACL72 | 0.70 | 0.55 | 23.10 | 0.23 | 3.15 | 0.38 |
| APL72 | 7.29 | 0^*^ | 0.51 | 0^*^ | 0.86 | 0.05 |
| EN100 | 3503.50 | 0.29 | 7165.10 | 0.01 | 3275.60 | 0.15 |
| NC100 | 939.97 | 0.49 | 437.03 | 0.04 | 649.64 | 0.34 |
| ACL100 | 0.41 | 0.23 | 7.17 | 0.27 | 1.53 | 0.36 |
| APL100 | 9.18 | 0.10 | 5.60 | 0.21 | 3.01 | 0.28 |

AFE: age at first egg, OP: oviposition period, ENX: cumulative egg number till X weeks of age, NCX: number of clutches till X weeks of age, ACLX: average clutch length till X weeks of age, APLX: average pause length till X weeks of age.

For Beijing-You chickens, standard errors ranged from 0.10 to 0.21 for heritabilitie. For White Leghorn chickens, standard errors ranged from 0.12 to 0.18 for heritabilities. For crossbreds, standard errors ranged from 0.08 to 0.14 for heritabilities.

* The corresponding variance component was close to 0.

**Table S12. Variances, heritabilities, and repeatabilities of egg quality traits for Beijing-You, White Leghorns, and crossbreds**

| **Trait** | **Beijing-You** | | | **White Leghorns** | | | **Crossbreds** | | |
| --- | --- | --- | --- | --- | --- | --- | --- | --- | --- |
|  | **Phenotypic variance (**$\boldsymbol{\sigma}_{\boldsymbol{p}}^{\boldsymbol{2}}$**)** | **Heritability (*h^2^*)** | **Repeatability (*r*)** | **Phenotypic variance (**$\boldsymbol{\sigma}_{\boldsymbol{p}}^{\boldsymbol{2}}$**)** | **Heritability (*h^2^*)** | **Repeatability (*r*)** | **Phenotypic variance (**$\boldsymbol{\sigma}_{\boldsymbol{p}}^{\boldsymbol{2}}$**)** | **Heritability (*h^2^*)** | **Repeatability (*r*)** |
| ESI32 | 8.08 | 0.26 | 0.44 | 8.77 | 0.12 | 0.37 | 6.76 | 0.36 | 0.45 |
| ESC32 | 60.78 | 0.49 | 0.71 | 12.75 | 0.20 | 0.21 | 48.79 | 0.44 | 0.67 |
| ESS32 | 0.41 | 0.30 | 0.56 | 0.32 | 0.16 | 0.37 | 0.40 | 0.27 | 0.53 |
| EST32 | 8.56E-04 | 0.29 | 0.44 | 7.10E-04 | 0.08 | 0.41 | 6.63E-04 | 0.31 | 0.52 |
| ESR32 | 0.54 | 0.49 | 0.57 | 0.41 | 0.10 | 0.48 | 0.47 | 0.29 | 0.57 |
| YR32 | 4.67 | 0.34 | 0.49 | 3.26 | 0.15 | 0.54 | 4.60 | 0.43 | 0.54 |
| YC32 | 1.82 | 0.06 | 0.43 | 1.43 | 0.11 | 0.42 | 1.58 | 0.17 | 0.45 |
| HU32 | 96.62 | 0.07 | 0.07 | 100.55 | 0.08 | 0.17 | 83.32 | 0.20 | 0.25 |
| ESI54 | 8.80 | 0.15 | 0.44 | 9.41 | 0.10 | 0.56 | 7.96 | 0.36 | 0.44 |
| ESC54 | 56.44 | 0.46 | 0.73 | 11.84 | 0.12 | 0.15 | 36.34 | 0.44 | 0.57 |
| ESS54 | 0.51 | 0.32 | 0.52 | 0.40 | 0.16 | 0.45 | 0.49 | 0.10 | 0.45 |
| EST54 | 8.62E-04 | 0.22 | 0.41 | 5.98E-04 | 0.22 | 0.42 | 7.10E-04 | 0.23 | 0.40 |
| ESR54 | 0.61 | 0.39 | 0.56 | 0.56 | 0.20 | 0.51 | 0.58 | 0.24 | 0.43 |
| YR54 | 4.94 | 0.53 | 0.78 | 2.74 | 0.04 | 0.68 | 3.56 | 0.53 | 0.74 |
| YC54 | 0.89 | 0.08 | 0.46 | 0.58 | 0.38 | 0.57 | 0.65 | 0.17 | 0.47 |
| HU54 | 46.65 | 0.20 | 0.47 | 33.29 | 0.08 | 0.54 | 51.21 | 0.46 | 0.60 |
| ESI72 | 10.51 | 0^*^ | 0^*^ | 14.64 | 0.05 | 0.47 | 9.66 | 0.27 | 0.46 |
| ESC72 | 53.94 | 0.31 | 0.67 | 12.41 | 0.05 | 0.05 | 37.98 | 0.53 | 0.64 |
| ESS72 | 0.74 | 0.29 | 0.62 | 0.57 | 0.14 | 0.46 | 0.54 | 0.11 | 0.40 |
| EST72 | 1.26E-03 | 0.19 | 0.60 | 1.09E-03 | 0.14 | 0.29 | 7.90E-04 | 0.08 | 0.36 |
| ESR72 | 1.33 | 0.13 | 0.39 | 1.07 | 0^*^ | 0.19 | 1.02 | 0.12 | 0.32 |
| YR72 | 7.35 | 0.21 | 0.47 | 3.52 | 0.22 | 0.53 | 4.25 | 0.35 | 0.68 |
| YC72 | 1.77 | 0.05 | 0.44 | 1.68 | 0.11 | 0.53 | 1.80 | 0.27 | 0.39 |
| HU72 | 102.25 | 0^*^ | 0^*^ | 49.95 | 0^*^ | 0.46 | 101.15 | 0.38 | 0.48 |
| ESI86 | 9.54 | 0.12 | 0.38 | 15.32 | 0.07 | 0.49 | 13.34 | 0.39 | 0.55 |
| ESC86 | 53.93 | 0^*^ | 0* | 7.26 | 0.17 | 0.17 | 30.05 | 0.45 | 0.74 |
| ESS86 | 0.88 | 0.07 | 0.54 | 0.72 | 0.30 | 0.41 | 0.78 | 0.20 | 0.48 |
| EST86 | 1.20E-03 | 0.20 | 0.42 | 1.18E-03 | 0.21 | 0.31 | 9.29E-04 | 0.11 | 0.36 |
| ESR86 | 0.97 | 0.38 | 0.50 | 0.90 | 0.15 | 0.40 | 0.93 | 0.10 | 0.32 |
| YR86 | 4.97 | 0.01 | 0.68 | 4.10 | 0.15 | 0.52 | 5.19 | 0.23 | 0.69 |
| YC86 | 2.81 | 0.14 | 0.67 | 2.49 | 0.23 | 0.65 | 2.51 | 0.25 | 0.58 |
| HU86 | 116.89 | 0.18 | 0.37 | 111.50 | 0^*^ | 0.11 | 162.15 | 0.35 | 0.47 |
| ESI100 | 17.28 | 0.14 | 0.42 | 17.48 | 0.31 | 0.51 | 13.29 | 0.26 | 0.49 |
| ESC100 | 51.09 | 0.21 | 0.78 | 10.35 | 0.21 | 0.35 | 28.62 | 0.30 | 0.70 |
| ESS100 | 0.73 | 0.06 | 0.52 | 0.69 | 0.09 | 0.43 | 0.70 | 0.19 | 0.51 |
| EST100 | 1.98E-03 | 0.13 | 0.13 | 1.70E-03 | 0.07 | 0.22 | 2.00E-03 | 0.04 | 0.19 |
| ESR100 | 1.68 | 0.08 | 0.22 | 1.59 | 0.14 | 0.23 | 1.36 | 0.11 | 0.24 |
| YR100 | 6.88 | 0.44 | 0.64 | 5.13 | 0^*^ | 0.38 | 6.46 | 0.28 | 0.62 |
| YC100 | 2.88 | 0.16 | 0.52 | 2.28 | 0^*^ | 0.64 | 2.86 | 0.28 | 0.65 |
| HU100 | 162.85 | 0.01 | 0.35 | 160.45 | 0.23 | 0.58 | 180.54 | 0.32 | 0.56 |

ESIX: egg shape index at X weeks of age, ESCX: eggshell colour at X weeks of age, ESSX: eggshell strength at X weeks of age, ESTX: eggshell thickness at X weeks of age, ESRX: eggshell ratio at X weeks of age, YRX: yolk ratio at X weeks of age, YCX: yolk colour at X weeks of age, HUX: Haugh unit at X weeks of age.

For Beijing-You chickens, standard errors ranged from 0.03 to 0.24 for heritabilities, and from 0.03 to 0.08 for repeatabilities. For White Leghorn chickens, standard errors ranged from 0.04 to 0.21 for heritabilities, and from 0.03 to 0.06 for repeatabilities. For crossbreds, standard errors ranged from 0.05 to 0.12 for heritabilities, and from 0.02 to 0.04 for repeatabilities

* The corresponding variance component was close to 0.
