# Supplementary material for: Genetic parameters, reciprocal cross differences, and age-related heterosis of egg-laying performance in chickens
Source: Genet Sel Evol. 2023 Dec 7;55:87. doi: 10.1186/s12711-023-00862-7 (PMC10702067; doi:10.1186/s12711-023-00862-7)
Supplement: Supplementary file 7 — Additional file 7: Table S13. Variances for egg weight traits at different ages with the univariate model. Table S14. Variances for egg quality traits at different ages with the univariate model. [file 12711_2023_862_MOESM7_ESM.docx]

**Additional file 7 Table S13-S14**

The variances for egg weight and egg quality with the univariate model are shown in Table S13 and S14, respectively.

**Table S13.** **Variances for egg weight traits at different ages with the univariate model.**

| **Traits** | **Additive variance (**$\boldsymbol{\sigma}_{\boldsymbol{a}}^{\boldsymbol{2}}$**)** | **Permanent environment variance (**$\boldsymbol{\sigma}_{\boldsymbol{pe}}^{\boldsymbol{2}}$**)** | **Residual variance (**$\boldsymbol{\sigma}_{\boldsymbol{e}}^{\boldsymbol{2}}$**)** |
| --- | --- | --- | --- |
| FEWt | 5.72 | 3.68 | 10.47 |
| EWt28 | 6.04 | 3.21 | 3.77 |
| EWt32 | 6.83 | 2.58 | 4.02 |
| EWt36 | 9.20 | 3.38 | 2.15 |
| EWt40 | 9.87 | 3.73 | 2.38 |
| EWt44 | 9.40 | 3.10 | 3.76 |
| EWt48 | 13.87 | 1.70 | 2.83 |
| EWt52 | 12.70 | 2.22 | 3.49 |
| EWt56 | 12.40 | 3.75 | 3.34 |
| EWt60 | 10.28 | 6.22 | 3.18 |
| EWt64 | 13.97 | 3.64 | 3.07 |
| EWt68 | 12.38 | 4.77 | 4.65 |
| EWt72 | 9.90 | 5.43 | 6.59 |
| EWt76 | 12.78 | 5.98 | 4.58 |
| EWt86 | 15.65 | 5.14 | 7.21 |
| EWt100 | 12.08 | 9.68 | 8.08 |

FEWt: weight for the first three egg, EWtX: egg weight at X weeks of age.

**Table S14. Variances for egg quality traits at different ages with the univariate model.**

| **Traits** | **32 weeks of age** | | | **54 weeks of age** | | | **72 weeks of age** | | | **86 weeks of age** | | | **100 weeks of age** | | |
| --- | --- | --- | --- | --- | --- | --- | --- | --- | --- | --- | --- | --- | --- | --- | --- |
|  | $\boldsymbol{\sigma}_{\boldsymbol{a}}^{\boldsymbol{2}}$ | $\boldsymbol{\sigma}_{\boldsymbol{pe}}^{\boldsymbol{2}}$ | $\boldsymbol{\sigma}_{\boldsymbol{e}}^{\boldsymbol{2}}$ | $\boldsymbol{\sigma}_{\boldsymbol{a}}^{\boldsymbol{2}}$ | $\boldsymbol{\sigma}_{\boldsymbol{pe}}^{\boldsymbol{2}}$ | $\boldsymbol{\sigma}_{\boldsymbol{e}}^{\boldsymbol{2}}$ | $\boldsymbol{\sigma}_{\boldsymbol{a}}^{\boldsymbol{2}}$ | $\boldsymbol{\sigma}_{\boldsymbol{pe}}^{\boldsymbol{2}}$ | $\boldsymbol{\sigma}_{\boldsymbol{e}}^{\boldsymbol{2}}$ | $\boldsymbol{\sigma}_{\boldsymbol{a}}^{\boldsymbol{2}}$ | $\boldsymbol{\sigma}_{\boldsymbol{pe}}^{\boldsymbol{2}}$ | $\boldsymbol{\sigma}_{\boldsymbol{e}}^{\boldsymbol{2}}$ | $\boldsymbol{\sigma}_{\boldsymbol{a}}^{\boldsymbol{2}}$ | $\boldsymbol{\sigma}_{\boldsymbol{pe}}^{\boldsymbol{2}}$ | $\boldsymbol{\sigma}_{\boldsymbol{e}}^{\boldsymbol{2}}$ |
| ESI | 2.34 | 0.70 | 4.38 | 1.54 | 1.92 | 4.54 | 1.94 | 2.66 | 6.46 | 3.78 | 2.69 | 6.45 | 4.03 | 3.09 | 8.08 |
| ESC | 15.84 | 11.93 | 15.16 | 11.04 | 8.70 | 14.26 | 14.06 | 7.57 | 14.57 | 7.32 | 13.36 | 9.77 | 5.47 | 13.66 | 9.10 |
| ESS | 0.10 | 0.10 | 0.19 | 0.09 | 0.14 | 0.25 | 0.08 | 0.20 | 0.31 | 0.18 | 0.21 | 0.41 | 0.08 | 0.26 | 0.36 |
| EST | 1.81E-04 | 1.61E-04 | 3.81E-04 | 1.42E-04 | 1.31E-04 | 4.25E-04 | 2.95E-05 | 3.52E-04 | 5.76E-04 | 1.91E-04 | 1.89E-04 | 6.78E-04 | 5.26E-05 | 2.87E-04 | 1.57E-03 |
| ESR | 0.16 | 0.11 | 0.21 | 0.16 | 0.13 | 0.30 | 0.08 | 0.27 | 0.76 | 0.17 | 0.18 | 0.57 | 0.12 | 0.22 | 1.15 |
| YR | 1.17 | 0.91 | 2.01 | 1.69 | 1.02 | 0.96 | 0.98 | 1.65 | 2.09 | 0.83 | 2.42 | 1.69 | 1.40 | 2.23 | 2.67 |
| YC | 0.19 | 0.58 | 0.89 | 0.15 | 0.17 | 0.36 | 0.31 | 0.39 | 0.99 | 0.46 | 1.13 | 0.98 | 0.53 | 1.17 | 1.05 |
| HU | 14.66 | 2.01 | 74.55 | 13.68 | 13.10 | 20.34 | 18.63 | 19.48 | 53.24 | 38.25 | 18.87 | 85.18 | 40.00 | 51.60 | 82.69 |

ESI: egg shape index, ESC: eggshell colour, ESS: eggshell strength, EST: eggshell thickness, ESR: eggshell ratio, YR: yolk ratio, YC: yolk colour, HU: Haugh unit.

$\sigma_{a}^{2}$: additive genetic variance, $\sigma_{pe}^{2}$: permanent environment variance, $\sigma_{e}^{2}$: residual variance
