# Supplementary material for: Genetic parameters, reciprocal cross differences, and age-related heterosis of egg-laying performance in chickens
Source: Genet Sel Evol. 2023 Dec 7;55:87. doi: 10.1186/s12711-023-00862-7 (PMC10702067; doi:10.1186/s12711-023-00862-7)
Supplement: Supplementary file 8 — Additional file 8: Figure S3. Heterosis of egg number at separate periods for reciprocal crosses. Figure S4. Heterosis of cumulative egg number for reciprocal crosses. [file 12711_2023_862_MOESM8_ESM.docx]

**Additional file 8 Figure S3-S4**

Heterosis of egg number at separate periods and cumulative egg number for reciprocal crosses are shown in Figure S3 and S4, respectively.

**WY**

**YW**

**Figure S3.** **Heterosis of egg number at separate periods for reciprocal crosses.**

**WY**

**YW**

**Figure S4.** **Heterosis of cumulative egg number for reciprocal crosses.**
