# Supplementary material for: Genetic parameters, reciprocal cross differences, and age-related heterosis of egg-laying performance in chickens
Source: Genet Sel Evol. 2023 Dec 7;55:87. doi: 10.1186/s12711-023-00862-7 (PMC10702067; doi:10.1186/s12711-023-00862-7)
Supplement: Supplementary file 9 — Additional file 9: Table S15. Predicted albumen height for the four genetic groups and heterosis for reciprocal crosses. [file 12711_2023_862_MOESM9_ESM.docx]

**Additional file 9 Table S15**

The predict values of albumen height and heterosis for reciprocal crosses are shown in Table S15.

**Table S15.** **Predicted albumen height for the four genetic groups and heterosis for reciprocal crosses.**

| **Traits** | **Genetic group** | | | | **H%(WY)** | **H%(YW)** | **Reciprocal cross differences (%)** |
| --- | --- | --- | --- | --- | --- | --- | --- |
|  | **WW** | **YY** | **WY** | **YW** |  |  |  |
| Alh32 | 5.00 | 4.24 | 4.53 | 4.46 | -1.9 | -3.6^**^ | 1.7 |
| Alh54 | 6.29 | 5.45 | 5.56 | 5.78 | -5.3^***^ | -1.6 | -3.7 |
| Alh72 | 6.03 | 5.42 | 5.37 | 5.63 | -6.1^***^ | -1.5 | -4.6 |
| Alh86 | 5.31 | 4.99 | 4.73 | 5.01 | -8.0^***^ | -2.7 | -5.3 |
| Alh100 | 5.15 | 4.86 | 4.53 | 4.87 | -9.6^***^ | -2.8 | -6.8 |

AlhX: albumen height at X weeks of age.

H%(WY): Percent heterosis for WY, the percentage to which the performance of WY is better than the average performance of the two parental lines, H%(YW): Percent heterosis for YW, the percentage to which the performance of YW is better than the average performance of the two parental lines.

Wald F statistics for H%(WY), H%(YW) and reciprocal cross differences were indicated as follows: **p*-value ≤ 0.05, ** *p*-value ≤ 0.01, *** *p*-value ≤ 0.001.
