# Supplementary material for: Genetic parameters, reciprocal cross differences, and age-related heterosis of egg-laying performance in chickens
Source: Genet Sel Evol. 2023 Dec 7;55:87. doi: 10.1186/s12711-023-00862-7 (PMC10702067; doi:10.1186/s12711-023-00862-7)
Supplement: Supplementary file 10 — Additional file 10: Figure S5. Spearman correlation coefficients between average heterosis and heritability for each trait. [file 12711_2023_862_MOESM10_ESM.docx]

**Additional file 10 Figure S5**

The relationship between average heterosis (absolute value) and heritability for each trait are shown in Figure S5. The relationship was estimated with the Spearman correlation coefficient using “ggpubr” R-package (<https://www.r-project.org/>). Across all detected traits, the observed correlation was not significant (correlation coefficient = -0.06, *p*-value = 0.65, Fig. S5a). For the three trait categories, a significant negative correlation (correlation coefficient = -0.64, *p*-value = 0.02, Fig. S5b) was only observed for the egg production traits.


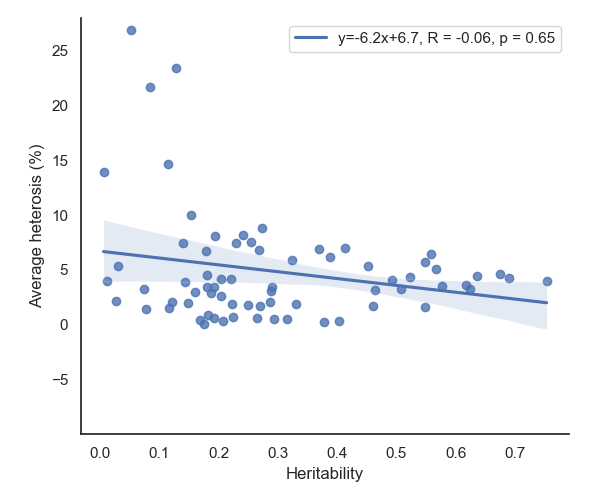

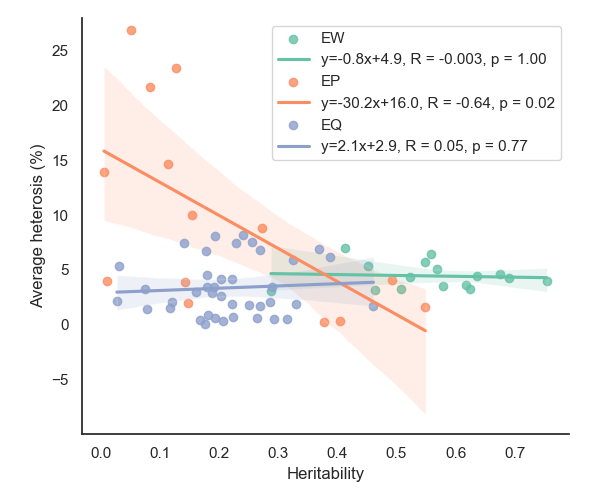


**a**

**b**

**Figure S5.** **Spearman correlation coefficients between average heterosis and heritability for each trait. a**: Spearman correlation coefficient between average heterosis (absolute value) and heritability for all the traits, **b**: Spearman correlation coefficient between average heterosis (absolute value) and heritability for each trait category. **EW**: egg weights, including egg weights at 16 timepoints; **EP**: egg production, including age at first egg, oviposition period, clutch related traits till 43, 72, and 100 weeks of age; **EQ**: egg quality, including egg shape index, eggshell colour, eggshell strength, eggshell thickness, eggshell ratio, yolk ratio, yolk colour, and Haugh unit at 32, 54, 72, 86, and 100 weeks of age.
